# Towards a quantitative description of nanostructured order in amorphous diamond-like carbon

**DOI:** 10.1038/s41467-026-77822-4

**Published:** 2026-09-17

**Authors:** Alan G. Salek, Hendrik Heimes, Ethan P. Turner, Edwin L. H. Mayes, Rebekka Klemmt, Jessica Wierbik, Xingshuo Huang, William R. Dunstan, Krysta-Leigh Douglass, Ryan Hogg, Nigel A. Marks, Espen D. Bøjesen, Jodie E. Bradby, Dougal G. McCulloch, Andrew V. Martin

**Affiliations:** 1 Department of Physics, School of Science, RMIT University, Melbourne, Victoria 3000 Australia; 2 Department of Materials Physics, Research School of Physics, The Australian National University, Acton, 2601 ACT, Australia; 3 Curtin University, Wark Ave, Bentley, 6102 Western Australia Australia; 4 RMIT Microscopy and Microanalysis Facility, RMIT University, Melbourne, Victoria 3000 Australia; 5 Center for Sustainable Energy Materials, Department of Chemistry and iNANO, Aarhus University, Langelandsgade 140, DK-8000 Aarhus C, Denmark

**Keywords:** Transmission electron microscopy, Structure of solids and liquids, Characterization and analytical techniques

## Abstract

Our knowledge of crystals rests on precisely measured structural models, which are lacking for amorphous matter. Without long-range order, the available experimental evidence matches competing structural models, including paracrystalline and random network models, limiting our understanding of formation mechanisms and properties. This impedes discovery of new amorphous materials, including high-density sp³-rich carbons, among the hardest known. We show that the pair-angle distribution function (PADF) overcomes these limitations, applying it to a non-crystalline sp³-rich carbon produced by compression of fullerenes at room temperature. The PADF reveals that this phase contains nanostructures with distinct short-range (4^th^–5^th^ neighbours, < 5 Å) and medium-range (5–10 Å) order. The nanostructure planes and angles relate to diamond, suggesting coordinated diamond-like tetrahedra too disordered to be nanodiamonds. The characterisation of nanoscale disorder via the PADF thus offers insight into the nature of structural order in amorphous materials and the formation pathways of diamond-like materials.

## Abstract (web summary)

Analysis of electron diffraction data shows that a hard amorphous carbon made by compressing fullerenes is not a random network, but contains nanoscale structures with diamondlike bonding that are too disordered to be nanodiamonds.

## Introduction

Materials with nanoscale disorder are a complex, yet technologically important, class of materials^1–3^. They are too disordered to be considered defective crystals, yet too ordered on the nanoscale to be considered truly amorphous. However, established structural characterisation methods, such as the pair-distribution function (PDF), do not conclusively determine whether materials are amorphous, or, in fact, have some nanoscale structural motifs or nanocrystalline domains^4^. The term amorphous masks heterogeneity and may represent a class of materials that can host synthesis-dependent nanostructures up to 1-2 nm in size^2^, such that nominally similar amorphous samples are not structurally equivalent^5^. Understanding disordered nanostructure is critical for applications in low-pressure crystal synthesis^6^ and nanocrystal synthesis from amorphous precursors^7^.

Crystalline materials are defined by their character of repetitive long-range atomic order, while amorphous phases are typically identified indirectly by the absence of experimental evidence for such crystallinity. This includes the lack of Bragg diffraction, the lack of resolvable crystal planes in transmission electron microscopy (TEM), and evidence from other techniques that are sensitive to structure, including nuclear magnetic resonance (NMR), Raman spectroscopy, and electron energy loss spectroscopy (EELS). The prevailing model for the structure of an amorphous material is a continuous random network (CRN), which can be generated algorithmically^8^. CRN-like models are also readily produced in molecular dynamics simulations^9,10^. However, for carbon, the existence of a true CRN is the subject of debate. Although several computational studies have constructed these CRN-like models, most recently via large-scale structural optimisation or thermodynamic sampling approaches^8,9^, there is little experimental evidence that a purely CRN is physically stable. Carbon readily reaches its lowest energy state by forming small sp^2^-bonded regions, which in turn prevents a true CRN from existing and differentiates it fundamentally from systems such as amorphous silicon, where only sp^3^ bonding exists. This raises the possibility that nominally ‘amorphous’ carbons may inherently have some character of nanoscale structural motifs and, hence, are not a true CRN.

Order beyond the 2^nd^ nearest neighbours is called medium- or intermediate-range order^11^. This typically starts at >5 Å for many materials but starts closer to ~3 Å for carbon because the second neighbour distance is ~2.5 Å. Medium-range order has been modelled with paracrystalline structures. However, the term paracrystalline has two distinct definitions in the literature: (i) a statistically distorted lattice where local displacements from the ideal lattice positions accumulate and destroy long-range order^10^, and (ii) very small ( ~ 10 Å) nanocrystals dispersed in an amorphous matrix^12,13^. Here, we use the term paracrystalline exclusively in the sense of describing small nanocrystals in an amorphous matrix. Nanostructures ( ~ 1-2 nm) have been reported in amorphous materials, including silicon and metallic glasses^12,14^. Paracrystalline models have been used to explain these experimental results^12,13,15–17^, challenging the paradigm that assumes amorphous materials are CRNs with no intermediate range order.

Carbon materials with a high fraction of diamond-like bonding ( > 80% sp^3^) form well-known crystalline and amorphous phases^18^. Diamond can exist in polycrystalline form with crystal diameters down to ~2 nm^19^. Even at these small sizes, polycrystalline diamond exhibits broadened Bragg diffraction and characteristic signatures in NMR, Raman spectroscopy, and EELS. Lattice fringes from nanodiamond crystals can be observed directly in TEM.

High-density sp^3^-bonded amorphous carbon phases can be grown as films^18,20,21^, by compression and laser-heating glassy carbon^22,23^ or, more recently, by compressing and annealing fullerene crystals^13,17,24^. These materials have a hardness and density approaching that of diamond, and sp^3^ fractions reported between 80–100 %^9,11,12,14,16,17^. Such amorphous carbons are referred to by various names including tetrahedral amorphous carbon^20^, amorphous diamond^22,23^ and paracrystalline diamond^13^. Beyond the residual presence of sp^2^ bonds, it is unclear if these high-density sp^3^-bonded carbons are distinct materials due to the presence of disordered nanostructure. In Tang et al.^13^ and Shang et al.^17^, TEM imaging was used to identify lattice planes of nano-sized crystals by observing their lattice fringe projections, providing evidence for non-amorphous structures in these materials that appeared amorphous by many standard characterisation methods. The paracrystalline structures were around ~1 nm in size, implying they contain tens or hundreds of atoms, but other structural details remain unknown and require new characterisation techniques. The extent and nature of structural order in these materials, and what it implies for their formation mechanisms and properties, remains poorly understood. It is critical that new characterisation techniques are based on a detailed statistical analysis of structure, which can infer the properties of the bulk material, and not the subjective analysis of individual TEM images. This motivates the development of characterisation techniques capable of probing statistically significant structure beyond average short-range order.

Fluctuation electron microscopy (FEM) and fluctuation diffraction are statistical techniques with a demonstrated sensitivity for probing medium-range order in amorphous materials^1,25^ and have been used to support paracrystalline models^12,15^. An extensive FEM study of several carbon materials by Zhao et al.^25^ showed the sensitivity of FEM to local structures in disordered graphitic carbons and fullerenes. Zhao et al. included a tetrahedral amorphous carbon in their study, which yielded a weak FEM signal interpreted as a CRN-like structure. Despite their demonstrated sensitivity, fluctuation microscopy and fluctuation diffraction are reliant on available models for structural interpretation and have not fully resolved structural debates concerning paracrystals and CRN structures^26^. They are yet to be widely adopted as a standard characterisation technique for amorphous materials. This motivates the development of new approaches to extract real-space structural information from fluctuation data.

The pair-angle distribution function^27,28^, PADF, is an emerging structural analysis technique for disordered or amorphous structures that measures three- and four-atom correlations. It contains angular information that is highly sensitive to the presence of nanostructures, even if they are not crystalline. The PADF is measured experimentally with nanobeam diffraction, and this type of data has a demonstrated sensitivity to nanostructure and/or medium-range order in many prior studies^1,12,29–31^. This overcomes the limitations of other methods such as projection TEM imaging, which rely heavily on paracrystalline or nanocrystalline models to detect nanostructure. It also overcomes the limitation of established pair distribution methods that lack local angular information. PADF measurements can be performed on TEMs with a nanobeam diffraction capability and it has similar coherence requirements to other standard electron diffraction techniques. The PADF technique has been used to study liquid crystals^32,33^, the short-range order of sp^2^-bonded activated carbons^34^, and the extended range order of sp^2^-hybridised disordered carbons^35^. Both the activated carbons and sp^2^-hybridised disordered carbons have locally graphitic structure that is clearly observable in high resolution TEM imaging, and neither is considered an amorphous carbon^36^. To date, there have been no reported PADF measurements of an amorphous material to our knowledge.

We recently reported a material produced by compressing C_60_ fullerene to 49 GPa under non-hydrostatic conditions at room temperature^37^. The recovered material contained narrow bands of nanodiamonds, suggesting a possible route to room-temperature diamond synthesis. However, most of the recovered sample was in a high-density sp^3^-rich ( ~ 85 %) phase that we previously classified as amorphous diamond-like carbon. This phase showed no evidence for long-range order or paracrystals with conventional TEM imaging and diffraction analysis. Here, we interrogate the amorphous structure in depth by measuring its PADF to determine if its structure is truly an amorphous CRN-like structure or, rather, a disordered nanostructured material. We compare these results to PADFs measured from the untransformed C_60_ crystals, the diamond band, and a separate nanodiamond sample^38^. Simulated PADFs from the ideal diamond structure and an MD simulation of pressure-induced changes to C_60_ were used to interpret the angular PADF information.

## Results

### Sample and PADF measurements

**Fig. 1:**
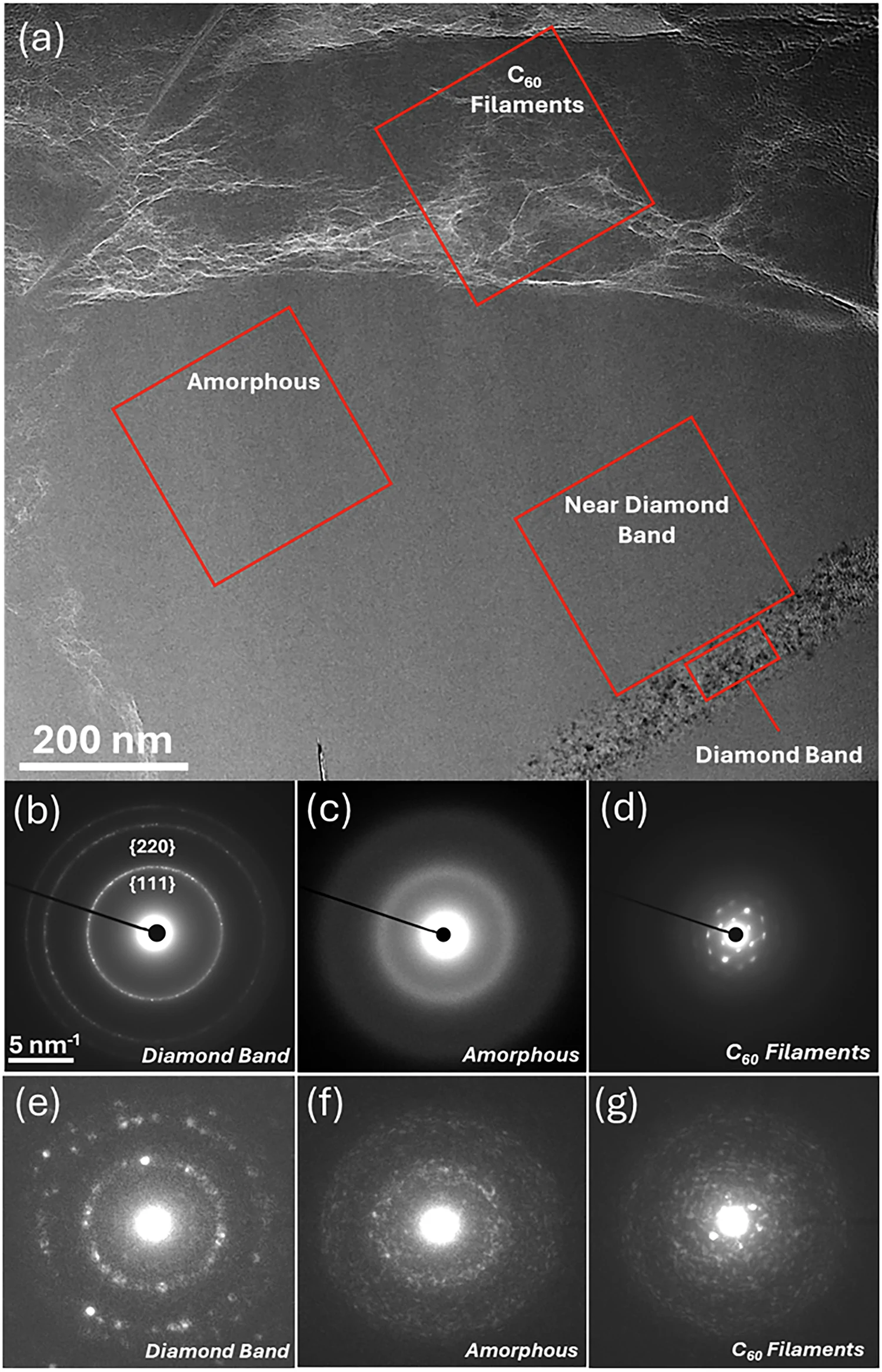
The compressed C_60_ sample. **a** A bright-field TEM image of the sample showing the approximate scan areas (red boxes) and locations of the amorphous regions, filaments and band of diamond crystals. Note the amorphous region is not the exact measurement location, but is indicative of the separation of the measured amorphous regions (labelled Amorphous Region 1 and Amorphous Region 2 in later figures) from the non-amorphous regions. Example selected area electron diffraction (SAED) patterns from **b** diamond band, **c** amorphous region, and **d** C_60_ filament region. The SAED patterns are produced from an aperture approximately 150 – 300 nm in diameter, which is a significantly larger beam size than the scanning nanobeam diffraction used for PADF measurements. Equivalent example nanobeam (NB) diffraction patterns, from a 0.8 nm probe, from **e** diamond band, **f** amorphous region, and **g** C_60_ filament region are shown. All SAED and nanobeam diffraction patterns are presented with the same scale.

Figure 1a shows a TEM image of the sample recovered from the diamond anvil cell revealing the amorphous phase as the predominant structure. There are bands of nanodiamonds and filaments streaking through the sample, and the boxes in Fig. 1a show indicative regions selected for further study. The diamond phase exhibits a sharp ring-like diffraction pattern consistent with randomly oriented nanocrystalline diamond (Fig. 1b), while the amorphous phase shows the characteristic broad diffraction typical of an amorphous or highly disordered structure (Fig. 1c). The diffraction from the filament regions contains peaks from intact C_60_ crystals overlapping smooth amorphous rings (Fig. 1d). The nanobeam diffraction patterns of these three regions, Fig. 1e–g, all show peaks or speckles that encode the local angular structure. As reported in Heimes et al.^37^, the amorphous phase in this sample showed no evidence of crystallinity.

PADF measurements were obtained from scanning electron nanobeam diffraction data. Using a JEOL JEM-F200 TEM, the electron beam was focused to 0.8 nm and scanned to collect 10,000 diffraction patterns. The PADFs were computed from the diffraction data using the *pypadf* Python package^28^. Further details are given in the Methods. Three different amorphous regions were measured to investigate the homogeneity of the amorphous phase, with their indicative positions indicated in Fig. 1a. Two of these regions are well separated from the filaments and diamond bands, while a third was measured closer (but not overlapping) a diamond band. Separate PADF measurements were made over the diamond band and the filaments. As a statistical technique, it is important to verify that the PADFs converge by comparing different subsets of the data. The convergence of the experimental PADFs is shown in the Supplementary Figs. 1-5. The PADF is a three-dimensional distribution of two pair distances *r*, *r’* and a relative angle θ between those pairs. Peaks in the PADF will be denoted by these three coordinates as $\left(r,{r}^{{\prime} },\theta \right)$, and we display the $r\mbox{'}=r$ cross-section because it provides information about all length scales and angles in a single 2D slice.

### The diamond band PADF

**Fig. 2:**
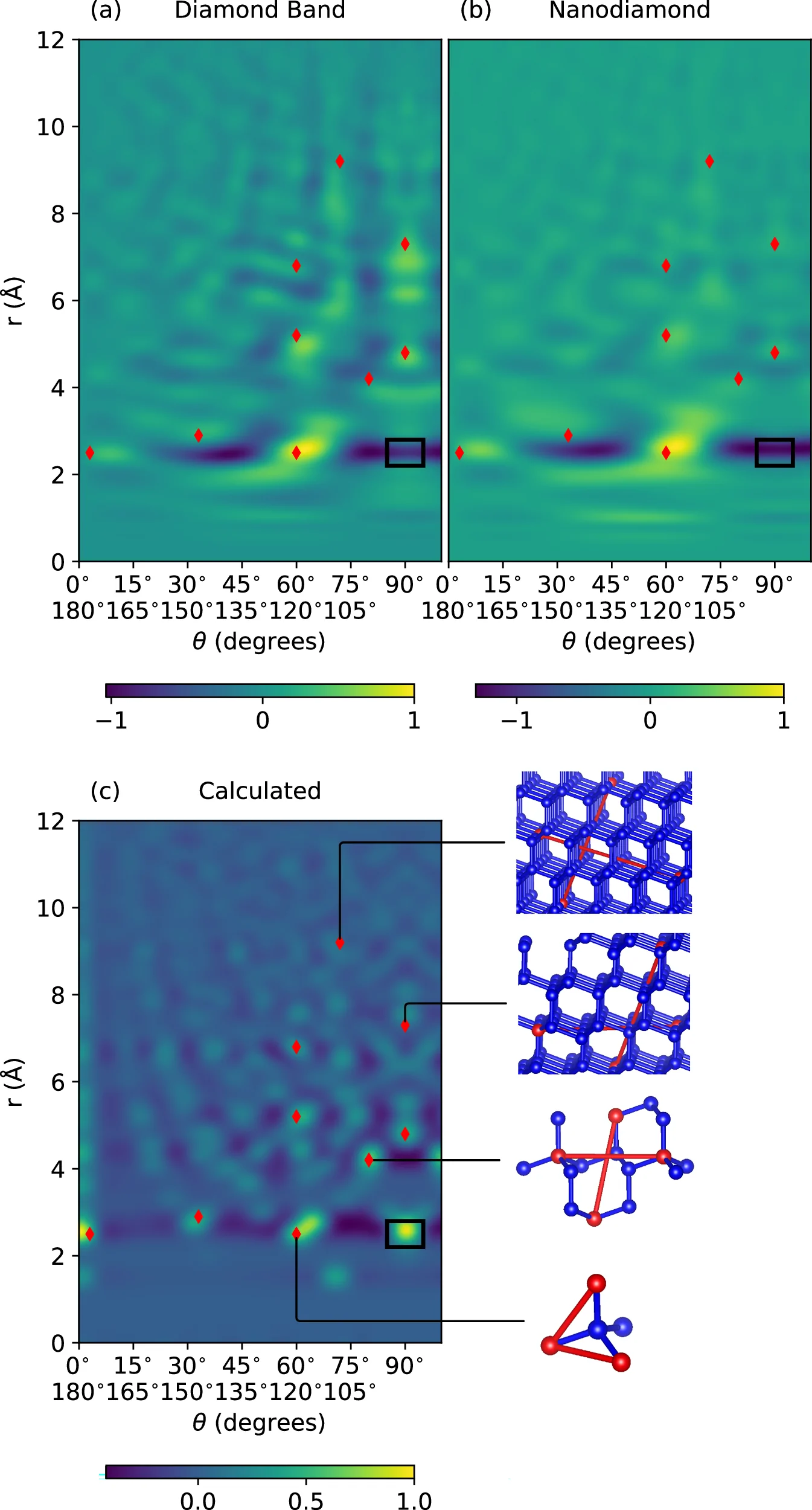
Diamond PADFs. **a** The $r=r\mbox{'}$ PADF slice of a diamond band in the compressed C_60_ sample. **b** A PADF of polycrystalline nanodiamonds fabricated by laser-heating glassy carbon^38^. **c** A simulated PADF from an ideal diamond structure. Selected peaks in the simulated structure are indicated by red markers, showing that peak locations match well to those observed in both datasets. Example pair combinations from the ideal diamond lattice that contribute to selected angular peaks are shown on the right-hand side of (**c**). All atoms in these models are C atoms in a diamond lattice and are therefore sp³-bonded; blue and red are used only to distinguish the pair of atoms contributing to the annotated peak (red) from the surrounding lattice (blue). The black square indicates a four-atom correlation that is weaker in the experimental data than in the model. The colour scale of each PADF was normalised separately to set the maximum value to 1 in the displayed slice.

Figure 2a shows the experimental PADF measured from a diamond band of the compressed C_60_ sample and compares it with a PADF of reference polycrystalline nanodiamonds^38^, shown in Fig. 2b. Both diamond results show a detailed pattern of peaks. The angular peak positions in both experimental diamond measurements match the PADF of an ideal model diamond structure well, depicted in Fig. 2c. The models shown in Fig. 2c present the hierarchy of structures that explain the PADF diamond peaks, including the second neighbour correlation at 2.5 ${{\mathrm{\AA}}}$, ring structures produced by 4^th^ and 5^th^ nearest neighbours at 4.2 ${{\mathrm{\AA}}}$, and finally ordered structures involving hundreds of atoms, spanning several unit cells at 7 ${{\mathrm{\AA}}}$ and beyond. There is a contrast difference between simulation and experiment, related to the finite experimental resolution or structural variations from the ideal crystal, which affects some peaks. For example, the black box in Fig. 2 shows a prominent 4-atom peak at 2.5 ${{\mathrm{\AA}}}$ and 90 ^o^ in the simulated diamond PADF, which is much smaller in the experimental PADFs and is obscured by the colour scale. There is further angular structure in the experimental data below 2 ${{\mathrm{\AA}}}$ which is not explained by the ideal diamond model, indicating improved resolution or improved structural models are required to explain the angular structure near the bond distance.

### The amorphous phase PADFs

**Fig. 3:**
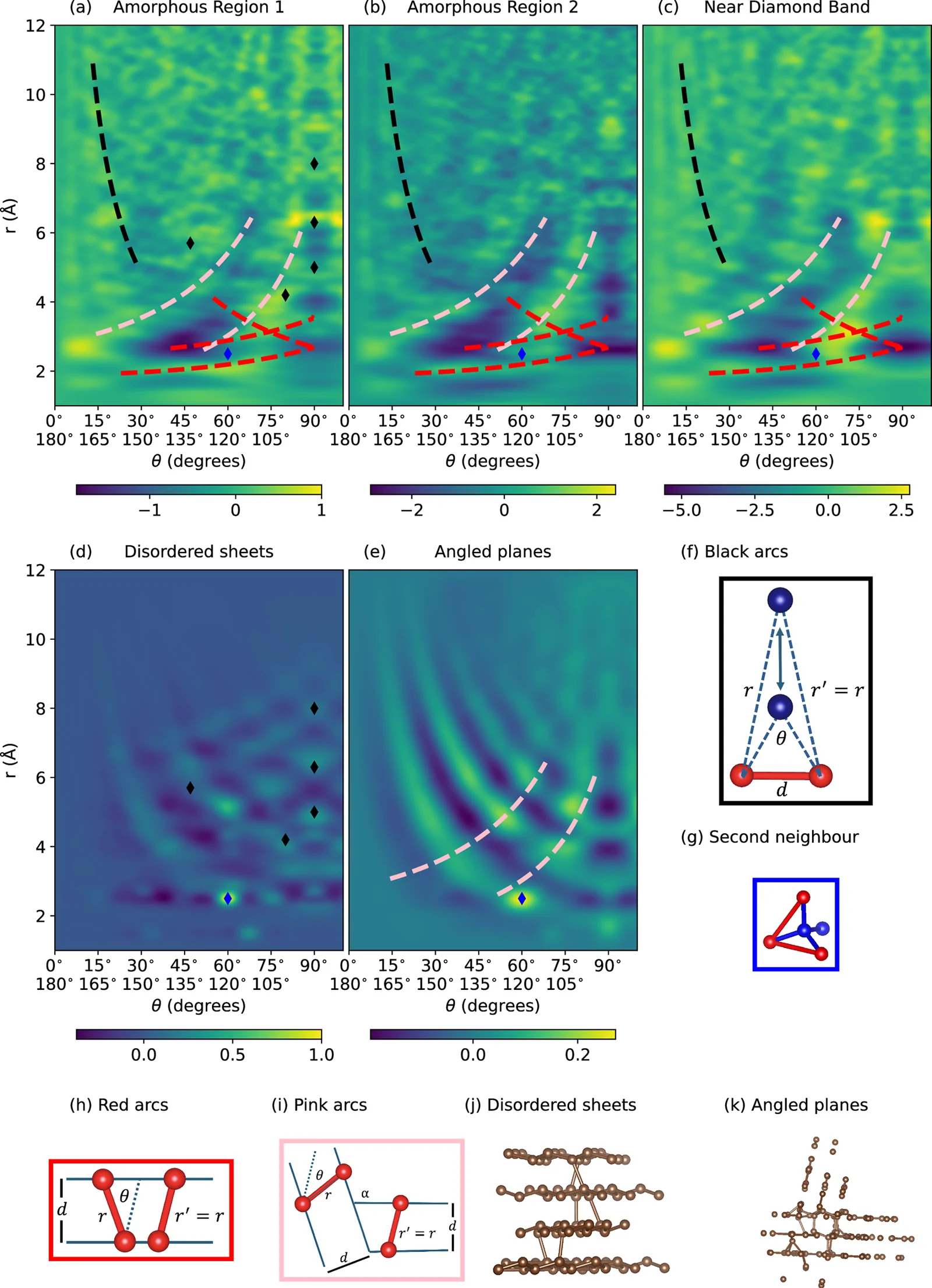
Amorphous Region PADFs. **a**, **b** The experimental *r = r'* cross-section of the PADFs for two different amorphous regions well separated from the diamond band, and **c** a third amorphous region close but not overlapping a diamond band. The colour scale of (**a**) was normalised to set the maximum value to 1, and (**b**, **c**) were put on the same colour scale. **d** The simulated PADF from randomly rotated and translated diamond layers (Disordered sheets model) and **e** from random atoms in planes at ${109.5}^{\circ }$ (Angled planes model). The colour scale of (**d**) was normalised to set the maximum value to 1, and the colour scale of (**e**) was put on the same colour scale. Structural models explain key features of the PADFs: **f** characteristic 2-atom pairs embedded in an amorphous structure give rise to the arcs indicated by black-dashed lines, **g** 2^nd^ neighbour correlation in an sp^3^ bond that gives rise to the annotated peak in blue diamond, **h** 4-atom correlations between two disordered planes give rise to red-dashed arcs, **i** 4-atom correlations arising from sheets at a fixed angle α, **j** the disordered sheets model that matches the angular structure of Amorphous Region 1 near ${90}^{\circ }$ indicated by the black diamonds, and **k** the angled planes model that better matches Amorphous Region 2 near ${90}^{\circ }$ and gives rise to the pink dashed arc. In the structural models of (**f**–**k**), all C atoms are drawn in blue and the indicative correlated pairs contributing to the annotated features are drawn in red; brown bonds in (**j**) and (**k**) are non-physical bonds introduced by the geometric construction of the model.

**Fig. 4:**
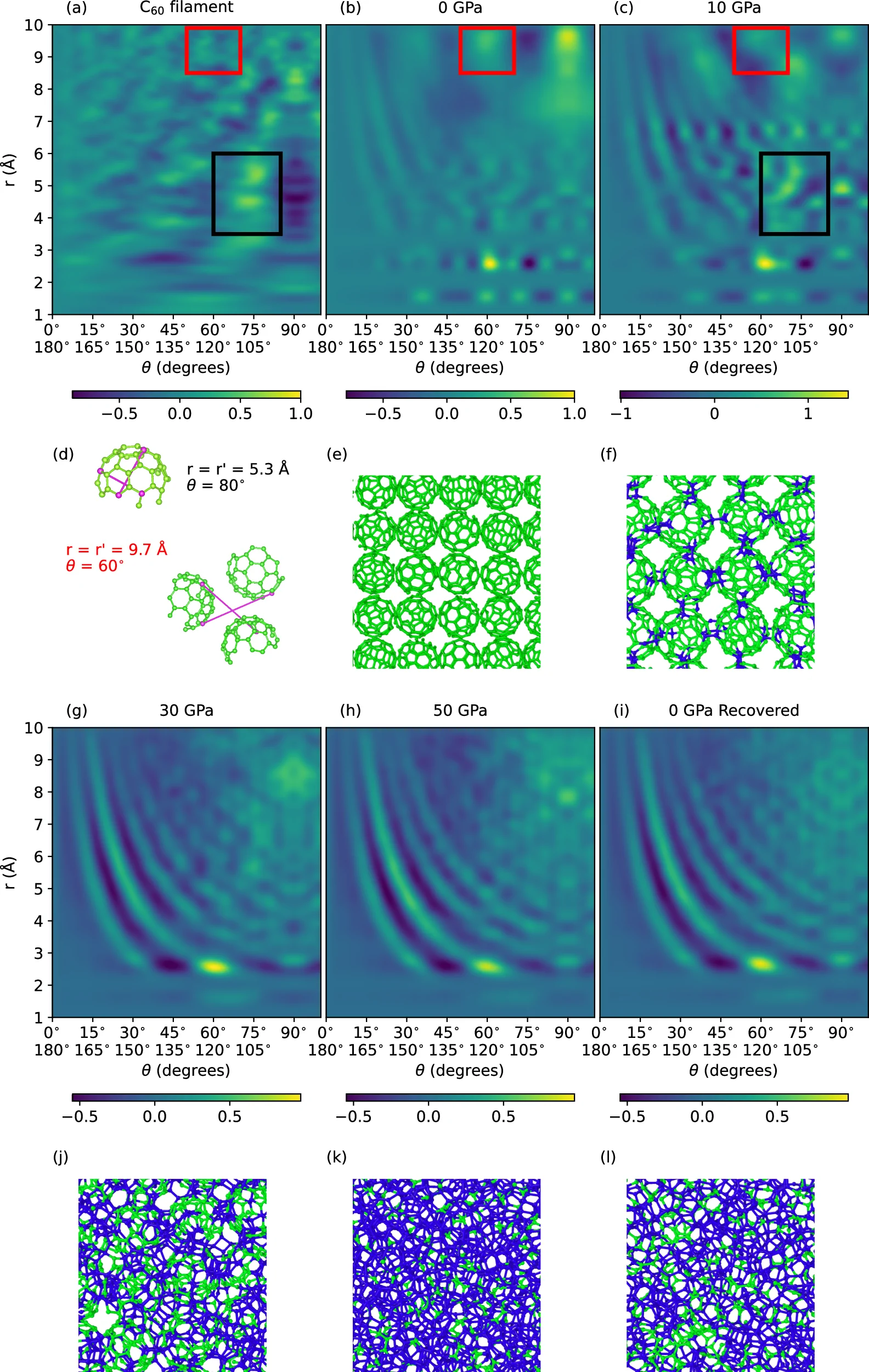
Filament region PADF and modelling. **a** Experimental PADF of a region with filament structures showing evidence of intact C_60_ cages. The colour scale of (**a**) was normalised to set the maximum value to 1. **a**–**c** and **g**–**i** Simulated PADFs of a C_60_ crystal under uniaxial compression. The colour scale of (**b**) was normalised to set the maximum value to 1, and (**c**), **g**–**i** were put on the same colour scale. **d** Examples models from a C_60_ crystal from the black and red regions showing key intra- and inter-cage correlations. **d**–**f**,** j**–**l** Models corresponding to the simulated PADFs with sp^3^ bonds shown in blue and sp^2^ bonds shown in green, which show how the molecular C_60_ cages become linked and then collapse. Indicative correlation pairs are shown in magenta.

Figure 3a–c show the PADF measurements of three amorphous regions of the sample, whose locations are marked in Fig. 1. In all three amorphous regions (Fig. 3a–c), the PADF has arcs in the range ${r} > \,4$ Å and$\,\theta \, < \,{45}^{\circ }$ that approach smaller angles at larger distances, as indicated by the black, dashed lines. We will refer to these as amorphous arcs, because they derive from characteristic pair distances, *d*, embedded in a random structure forming a 3-atom isosceles triangle^39^, indicated by Fig. 3f. As the third atom is randomly placed at different distances from the pair, enhanced correlations appear along the black, dashed arc according to the formula $r=d / \left(2 \sin \left(\theta / 2\right)\right.$. The black, dashed arc shown in Fig. 3a–c is for the distance *d* = 2.5 Å, which is close to the expected second-neighbour distance for both sp^2^ and sp^3^-bonded atoms. Further amorphous arcs are observed close to other prominent distances in diamond *d* = 1.5 Å and 4.2 Å and close to the expected distance between planes cross-linked by sp^3^ bonds, 1.9 Å (not annotated). When the structure approaches a random network, these arcs become strong, smooth and dominate the PADF (see Fig. 3e or Fig. 4g–i), because the third atom in the triangle (blue atom in Fig. 3(f)) can appear at any distance from the other two atoms. In the three experimental PADFs in Fig. 3, these arcs are present, suggesting some random character, but they are neither the strongest nor the most dominant feature of the PADF. This is evidence against a purely CRN explanation of these amorphous regions.

A PADF peak associated with second-nearest neighbour atoms occurs near ($2.5\,{{{\AA }}}$, $2.5\,{{{\AA }}}$, 60^o^), which is caused by the vertices of the sp^3^-bonded tetrahedral units (see the blue marker in Fig. 3a–c and diagram in Fig. 3g). Since the second neighbour peak of sp^2^-bonded planes is in a similar position, residual sp^2^ bonding from deconstructed fullerenes may contribute to this indicated peak. Hence, this peak does not discriminate between sp^2^- and sp^3^-bonded structures.

In the range r<5 Å, the PADFs of the three amorphous regions (Fig. 3a–c) exhibit strong arcs that approach larger angles at larger distances, as indicated by the dashed red and dashed pink lines. These arcs are not predicted by CRN-like models and we will refer to them as nanostructural arcs, because they indicate local non-random structure. The dashed red arcs are consistent with four-atom correlations generated by two disordered planes at fixed distances of 1.9 Å and 2.5 Å (see Fig. 3h). These red-dashed arcs follow the formula, $r=d/\cos (\theta /2)$. These distances indicate planes cross-linked by sp^3^ bonds.

In the intermediate order range, r>5 Å and θ>45^o^, the three amorphous regions show the largest variation in angular structure. This shows that the structure of the amorphous region is neither homogeneous on this length scale nor a random network. The characteristic length scales implied by these correlations correspond to nanostructural motifs of tens to hundreds of atoms large. The intermediate range order in Amorphous Region 1, indicated by the black dots in Fig. 3a, matches the angular structure predicted by randomly rotated and translated diamond layers with the $\{111\}$ diamond layer distance of 2.15$\,{{{\AA }}}$, shown in Fig. 3d, j. The agreement is particularly close for peaks at 90 °. The intermediate range order of all three amorphous regions, shown in Fig. 3a–c, has features similar to the pink, dashed arcs produced by planes at an angle of 109.5^o^, shown in Fig. 3e, i. The correlations along the pink dashed arc are predicted by the angled planes model with in-plane disorder, shown in Fig. 3k, and plane separations of 1.9 Å and 3.4 Å. The pink arc follows the formula is $r=(d{\sin }^{2}\frac{\alpha }{2})/(\sin \frac{{{\rm{\theta }}}-{{\rm{\alpha }}}}{2})$, which is derived from the angled planes model. We note, however, that the random distribution of atoms in the plane over-predicts the strength of the amorphous arcs. Although the disordered sheets and angled planes models are not physical atomic structures, they are useful for identifying geometric motifs.

Only some of the PADF peaks produced by diamond agree with those observed in the PADFs of the amorphous regions. This indicates that the intermediate-range order in the amorphous regions is not well explained by the presence of fully ordered nanocrystals. To further investigate this, PADFs were calculated from crystallisation models produced by annealing amorphous diamond models (see Supplementary Fig. 6). As the fraction of diamond increases in the models, the calculated PADFs show a growing contribution from the expected diamond PADF, but not the type of angular correlation observed in the experimental amorphous regions.

### The filament region PADF and modelling

Figure 4a shows the experimental PADF measured from a C_60_ filament within the sample. Distinct correlations are present in the vicinity of $4{{{\mathrm{\AA}}} } < {r} < \,6{{{\mathrm{\AA}}} }$ and ${60}^{o}\, < \theta \, < \,{80}^{o}$, indicated by the black box in Fig. 4a. PADFs computed from simulations of the uniaxial pressure experiment (Fig. 4b, c, g–i), show a similar angular structure at low pressures ( ~ 10 GPa), indicating this feature is due to intact C_60_ cages. An example of an intra-cage correlation contributing to this PADF peak is shown in Fig. 4d. Before any pressure is applied, intra-molecule correlations are prominent at short distances and inter-molecular correlations are associated with larger r values (i.e., $r=9\,{{{\AA }}}$ and θ=60^o^, as shown by the red box in Fig. 4a–c). These intercage correlations are expected from the lattice structures in the atomic models shown in Fig. 4e, f. As the pressure is increased, the amorphous arcs strengthen and a shift is observed in this $\left(9\,{{{\AA }}}\right.,60$^o^) peak to lower distances due to non-ordered interlinking of C_60_ molecules. Beyond 30 GPa, the inter-molecular PADF correlations are no longer evident, and the atomic models indicate that the molecular cages collapse, as shown in Fig. 4j–l.

## Discussion

The combined evidence from the experimental and simulated PADFs shows that the amorphous region of our compressed C_60_ sample is a diamond-like-bonded material with nanostructural (non-random) disorder; i.e., it does not conform to a purely random network structure and is too disordered to be genuine nanocrystals. If the intermediate-range order of the amorphous regions were nanocrystalline, the PADFs of Fig. 3 would agree more closely with those of Fig. 2. If the amorphous regions were CRN-like, they would agree more closely with CRN-like model PADFs such as Fig. 4h, i. An ideal CRN model is expected to have similar or less angular order to the simulated structures in Fig. 4h, i, including the 1^st^ and 2^nd^ neighbour peaks, the amorphous arcs, and a lack of medium-range order.

Different regions of the amorphous sample showed a common short-range order up to 4^th^ to 5^th^ nearest neighbours, and evidence of amorphous structure. The observed nanostructural PADF arcs indicate disordered planar motifs related to the {111} spacing of diamond or cross-linked planes, but with substantial disorder. A key question is the role of sp^2^ and sp^3^ bonds in these disordered nanostructures. Since their PADF correlations do not resemble graphene or nanoscale graphite, they are unlikely to be purely sp^2^ bonded. It is possible that hybridisation of sp^2^ and sp^3^ bonds is key to understanding their disordered nature, which could be quantified by further modelling of the nanostructures that agree with the experimental PADF measurements.

It is well known that diamond can exhibit stacking disorder of the {111} planes in the form of random cubic and hexagonal stacking. However, this type of stacking disorder alone does not account for the PADFs of the amorphous region (see Supplementary Fig. 7). A better agreement with experimental PADFs is achieved by adding substantial rotational or translational disorder (Disordered sheets model) or in-plane disorder (Angled planes model). Although these models are not the result of a physical simulation, they do capture geometric motifs in the sample.

Planar nanostructures may be consistent with the planar structures observed in TEM imaging that Tang et al.^13^ cited as evidence for paracrystals. The synthesis of Tang et al.’s material involved annealing to around 1600 K, whereas we used room temperature compression; hence, the structures of the two materials may differ. The PADF is complementary to TEM imaging and could constrain the degree of order in proposed paracrystalline models.

Beyond sp^3^-rich amorphous carbon, there are opportunities to apply the PADF to carbon materials with high sp^2^-bonding fractions. An earlier study of disordered carbons using FEM provided statistical evidence in *q*-space of intermediate range order that was attributed to structures at least 1 nm in size^25^. The FEM signal is part of the intensity correlation function that we compute en route to the PADF. There may be opportunities to study similar structures with the PADF. An early PADF study detected defects in an activated carbon^34^ but was limited to structures 3 ${{\mathrm{\AA}}}$ in size. Improvements in the PADF analysis method^28^ have enabled us to overcome this limitation to achieve the observation of intermediate range order presented here. Although the current focus of this work is amorphous carbons, the same PADF protocol is readily transferable to other amorphous systems (e.g., amorphous silicon), where variable structural motifs may be present. Beyond bulk materials, there are further opportunities to apply the PADF technique to complex nanoparticle structures such as carbon dots^40^.

There are opportunities to access larger distance scales in the PADF by increasing the probe size beyond the 0.8 nm used in this study. Our results show structural information up to 1.2 nm, suggesting that signal is measured from the outer parts of the beam or some spreading of the probe in the sample. Increasing the beam size increases the largest r value accessible in the PADF, but also creates a larger interaction volume which may require more diffraction data for convergence. It is unknown how the number of patterns will scale with probe size. The PADF is complementary to existing statistical measures, such as variable-resolution FEM that collect FEM signals as a function of probe size^41^. The comparison of PADF and variable-resolution FEM would corroborate both statistical significance and structural interpretation.

Overall, our results highlight the power of the PADF technique to resolve debates about the random network hypothesis and the structure of amorphous materials. It contains key angular information in real space that is not accessible by conventional electron or X-ray diffraction, PDF analysis, or spectroscopy. By quantifying the length scale and distribution of angular correlations, the PADF distinguishes random networks from other disordered structures. In addition, it provides new quantitative information to create improved models of disordered atomic structure. The PADF technique has future applications to glasses, metallic glasses, and other amorphous solids, which could lead to a fundamentally deeper understanding of amorphous structure and its relationship to material properties. Beyond characterisation, the physical insight gained through PADF analysis can directly inform synthesis strategies. In the present case, the rotational and angular correlations revealed by the PADF further confirm that non-hydrostatic compression, or shear conditions, favor the formation of diamond-like tetrahedral coordination networks. This points toward concrete experimental pathways for the targeted synthesis of new amorphous diamond-like materials.

## Methods

### Sample preparation

A Boehler-Almax plate diamond anvil cell (DAC) fitted with Almax type IIa diamonds (300 µm culets) was used to compress C₆₀ powder with micrometre-sized grain (99.5% pure, Sigma Aldrich). No pressure medium was used, so the sample was compressed under non-hydrostatic conditions. The gasket was stainless-steel, pre-indented to 50 + − 5 µm, with a sample chamber of 100–150 µm laser drilled. The sample was loaded and compressed at room temperature to a maximum pressure of 49 GPa, with pressure determined by the diamond Raman edge. The recovered sample was extracted from the DAC and TEM lamellae were created via Ga^+^ ion FIB-SEM milling and attached to a TEM grid. Compression without a pressure medium follows the approach of McCulloch et al.^42^ and Lai et al.^43^, and this specific sample is one of those characterised in Heimes et al.^37^.

The nanodiamond reference sample was prepared by loading glassy carbon (Hochtemperatur-Werkstoffe, Sigradur-G) into a DAC with an Ar pressure medium and compressing to 24 GPa at room temperature. Pressure was determined by the Raman diamond-line. The sample was then flash laser-heated with a Yttrium Lithium Fluoride (YLF) laser at 1070 nm delivered as a single high-power 40 ms square-wave pulse. Temperature was measured by imaging the centre of the laser spot through a pinhole and fitting the recorded grey-body emission spectrum with a Planck function, giving a peak temperature of 2700 K with an uncertainty of approximately ±100 K; heating and cooling times are on the order of 1 ms and 1–2 ms respectively, so this peak temperature is held for the duration of the flash^44^. The heated region was allowed to cool while still under pressure before decompression to ambient conditions. The recovered material was prepared for TEM by FIB-SEM. This preparation follows the approach of Cook et al.^44^.

### Data collection

Scanning electron nanobeam diffraction data was collected on a JEOL JEM-F200 TEM operated at 200 keV with a spot size of 0.8 nm and a convergence angle of 1.5 mrad. A 10 µm condenser lens aperture was used. Five 0.1 s exposures were taken at each probe position and summed. During each scan, the initial current was around 14–15 μA, which fell to around 11–12 μA by the end of the scan. Diffraction data was recorded on the Gatan Rio 16 CMOS camera with 4096 × 4096 pixels. All datasets were scanned at 100 × 100 probe positions at 3 nm spacing, except for the diamond band which was recorded at 1 nm spacing over 100 × 50 probe positions. The effective detector distance was 300 mm, giving a q value of 1.27 nm^−1^ at the edge of the detector.

### PADF analysis of the diffraction data

The PADF is an atomic correlation function that includes contributions from three- and four-atom correlations. It is a function of two pair distances (*r* and *r’*) and an angle between the pairs (θ). It is computed from the angular cross-correlation function of a scanning nanobeam diffraction dataset. The mathematical details of the intensity correlation function^45,46^, the PADF^27^ and the transformation to produce the PADF^28^ can be found in the cited literature. The collected data was rebinned to 1024 × 1024 pixels to reduce the data size. Then the data was centered by computing the cross-correlation function between each diffraction pattern and the first pattern. After centreing, the data was rebinned further to 256 × 256 pixels and analysed using the *pypadf* package^28^. A mask was applied to exclude horizontal and vertical detector artefacts. An angular intensity correlation function was calculated from the masked diffraction patterns, using 512 radial (q) bins and 256 angular bins. A difference correlation was computed to reduce the effects of background scattering^28^. The intensity correlation functions were masked to remove low *q* artefacts. The PADFs were computed from the masked intensity correlation functions using spherical harmonic functions from l=4 to l=64. The PADFs had 400 radial bins and 256 angular bins with a maximum radial distance computed of 16 ${{{\AA }}}$ and an angular range from 0 to 2π.

### PADF calculations from atomic models

PADFs were calculated from atomic models using the *model_padf.py* code (https://github.com/jack-binns/model_padf.git). The calculation first computes a 3D pair distribution on a grid with 48 radial bins, 360 polar angle bins and 720 azimuthal angle bins with a maximum radial value of 1 nm. A spherical harmonic expansion is then taken of the 3D pair distribution with harmonic orders from l=4 to l=64 to match the experiment. The *r*-dependent spherical harmonic coefficients were used to calculate the PADF via a weighted sum over Legendre polynomials (see Martin et al.^27,28^ for further mathematical details). The final PADF was computed on 48 radial bins to a maximum r value of 10 ${{{\AA }}}$, and 360 theta bins over the range 0−2π. All PADFs were multiplied by a $|\sin \theta |$ term to correct for angular sampling effects as described in Martin et al.^28^, except for the diamond simulation in Fig. 2 where the term was omitted to better visualise peaks near θ = 0 and 180^o^.

The diamond lattice models were constructed by tiling the diamond unit cell with a custom Python script. The hexagonal crystal (Space group: *P*6_3_/mmc; a = 2.52 Å, c = 4.17 Å) and the diamond crystal simulation used 9 × 9 × 9 unit cells. The stacking disorder model calculation used 20 separate crystals, each with 7 layers and 6 in-plane unit cells across. Each layer of the stacking disorder model was randomly added in a cubic or hexagonal geometry with 50% probability.

The C_60_ pressure simulations were performed using LAMMPS as previously described in Heimes et al.^37^. The simulation atomic volumes were cropped into a regular array of spheres of 15 ${{{\AA }}}$ radius and PADFs of each sphere were calculated independently, then averaged. This cropping was performed to remove low angular frequency artefacts from long-range four-atom correlations that occur with highly disordered atomic models.

## Supplementary information

Supplementary Information

Transparent Peer Review file

## Data Availability

The diffraction data and computed PADF files that support the findings of this study are available from the following FigShare repository: https://doi.org/10.25439/rmt.33201612.

## Code availability

Code to compute PADFs from diffraction data and atomic models is available at https://github.com/amartinrmit/pypadf (https://doi.org/10.5281/zenodo.21896047). The details of the code are described in Martin et al.^28^.
