# Supplementary material for: Towards a quantitative description of nanostructured order in amorphous diamond-like carbon
Source: Nat Commun. 2026 Sep 17;17:10938. doi: 10.1038/s41467-026-77822-4 (PMC13680357; doi:10.1038/s41467-026-77822-4)
Supplement: Supplementary file 1 [file 41467_2026_77822_MOESM1_ESM.pdf]

Supplementary Information: Towards a quantitative description of nanostructured order in amorphous diamond-like carbon

Alan G. Salek[1], Hendrik Heimes[2], Ethan P. Turner[3], Edwin Mayes[4], Rebekka Klemmt[5], Jessica Wierbik[2], Xingshuo Huang[2], William R. Dunstan[1], Krysta-Leigh Douglass[1], Ryan Hogg[1], Nigel A. Marks[3], Espen Bøjesen[5], Jodie E. Bradby[2], Dougal G. McCulloch[1,4], Andrew V. Martin[1].

1. Department of Physics, School of Science, RMIT University, Melbourne, Victoria 3000, Australia.
2. Department of Materials Physics, Research School of Physics, The Australian National University, Acton 2601, ACT, Australia.
3. Curtin University, Wark Ave, Bentley, 6102, Western Australia, Australia.
4. RMIT Microscopy and Microanalysis Facility, RMIT University, Melbourne, Victoria 3000, Australia.
5. Center for Sustainable Energy Materials, Department of Chemistry and iNANO, Aarhus University, Langelandsgade 140, DK-8000 Aarhus C, Denmark.

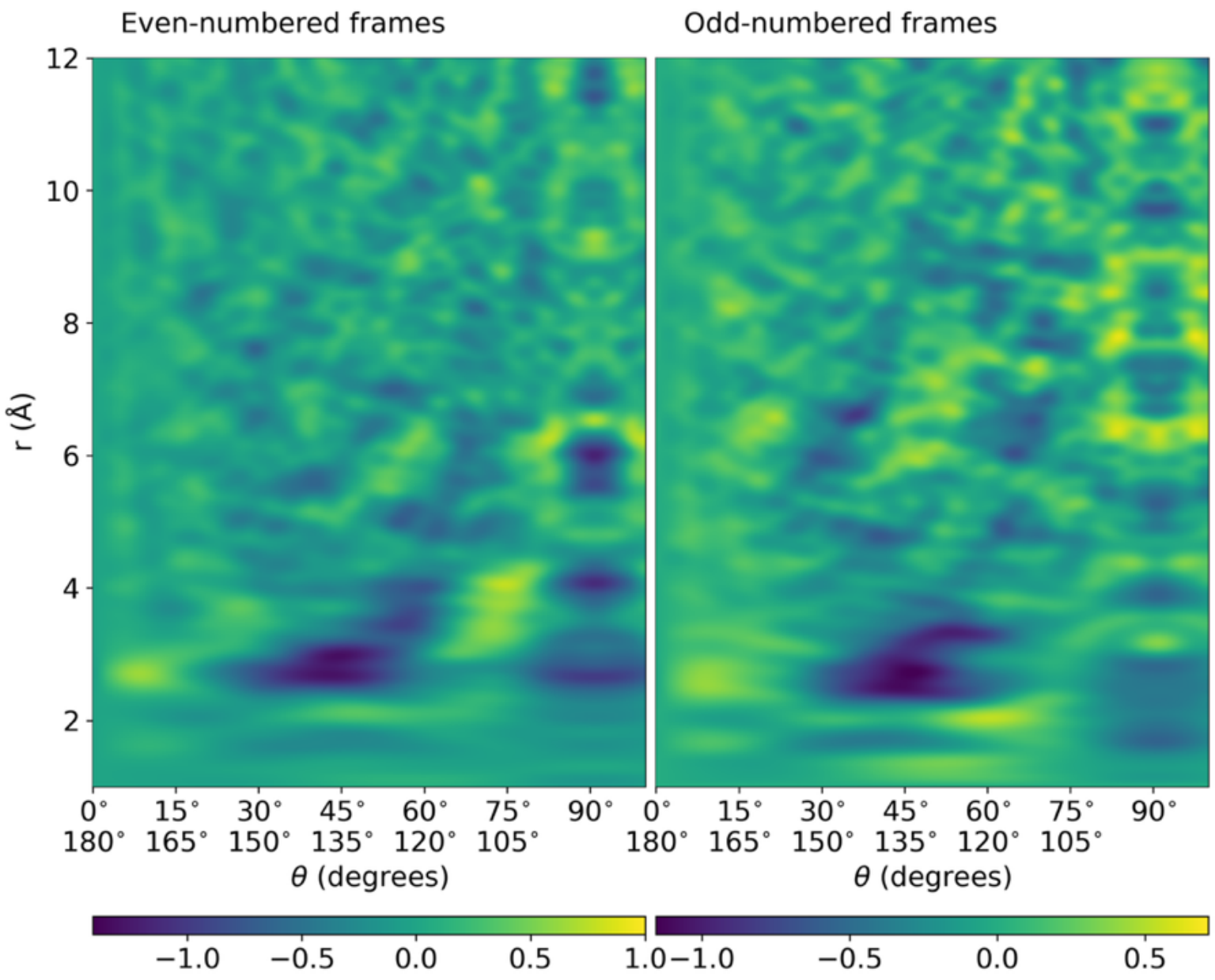

Supplementary Figure 1: Convergence test for Amorphous Region 1. The $r = r'$ slice of the PADF of Amorphous Region 1 calculated from the odd and even frames in the scan. Convergence is assessed visually by the location (r, θ) of positive PADF signals, but not by quantitative comparison of the colour scale. A sufficient level of convergence to identify amorphous arcs and nanostructural arcs was achieved, and the convergence decreases at larger r values.

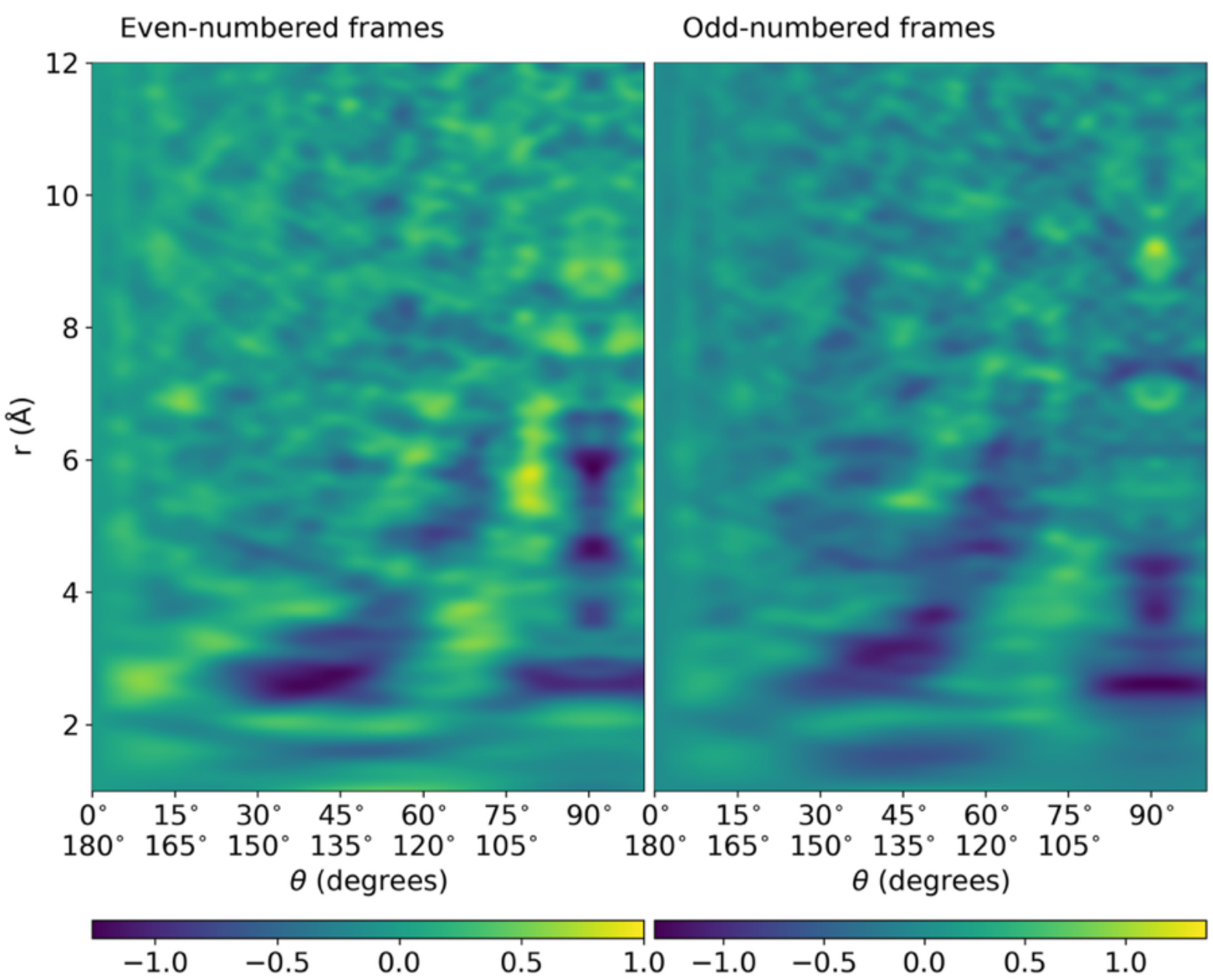

Supplementary Figure 2: Convergence test for Amorphous Region 2. The $r = r'$ slice of the PADF of Amorphous Region 2 calculated from the odd and even frames in the scan. Convergence is assessed visually by the location (r, θ) of positive PADF signals, but not by quantitative comparison of the colour scale. A sufficient level of convergence to identify amorphous arcs and nanostructural arcs was achieved, and the convergence decreases at larger r values.

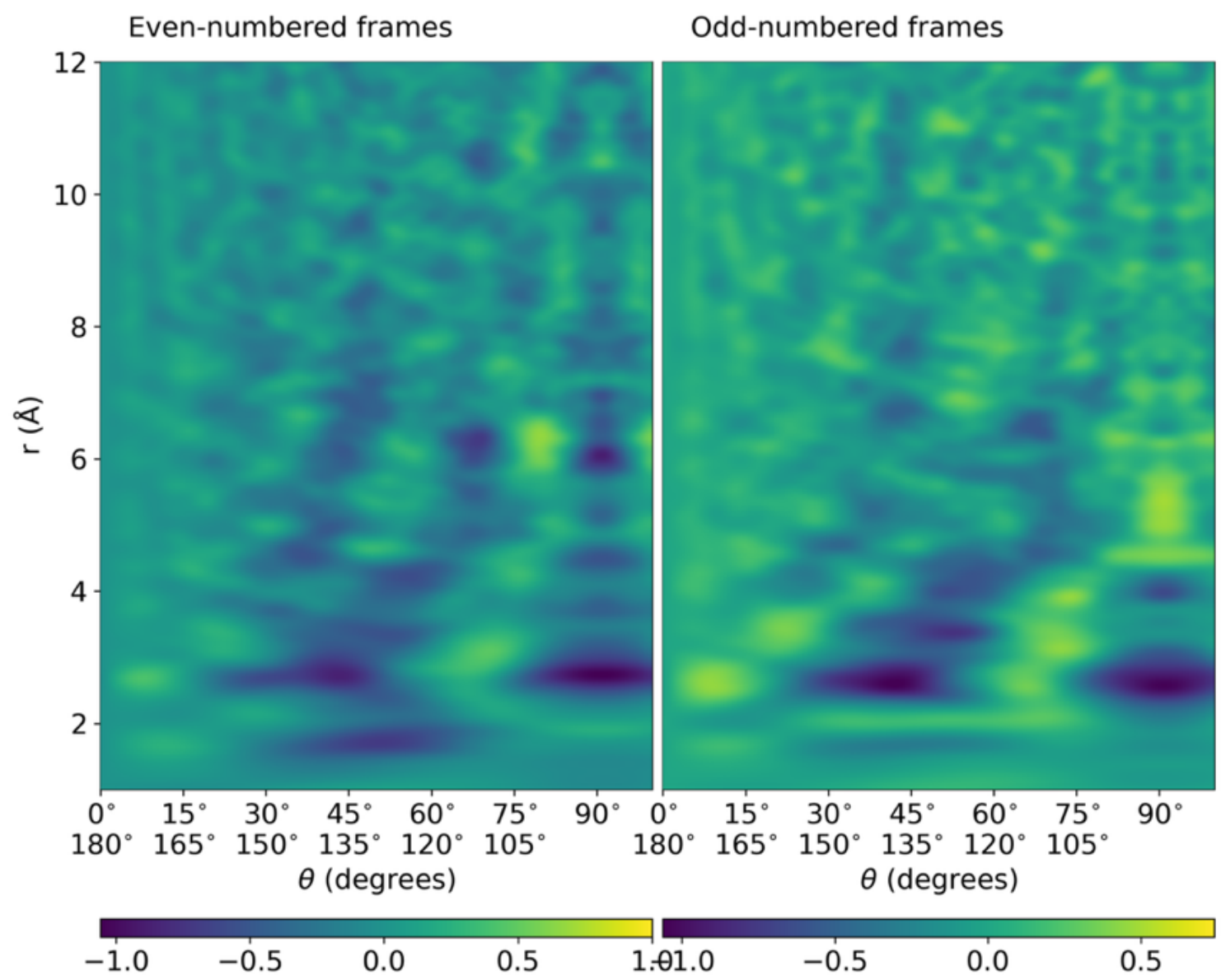

Supplementary Figure 3: Convergence test for the amorphous region near the diamond band. The $r = r'$ slice of the PADF of the amorphous region near the diamond band calculated from the odd and even frames in the scan. Convergence is assessed visually by the location (r, θ) of positive PADF signals, but not by quantitative comparison of the colour scale. A sufficient level of convergence to identify amorphous arcs and nanostructural arcs was achieved, and the convergence decreases at larger r values.

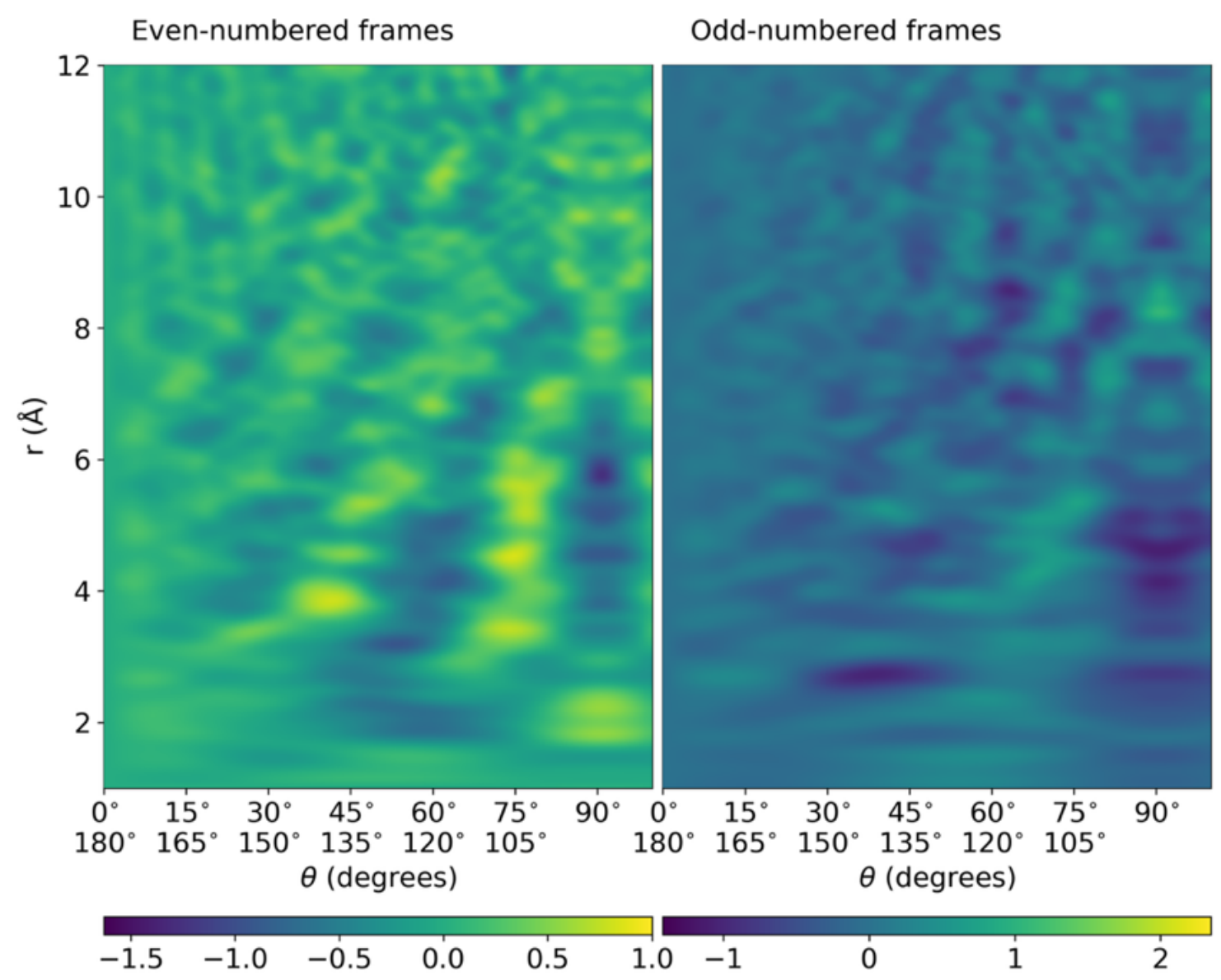

Supplementary Figure 4: Convergence test for a filament region. The $r = r'$ slice of the PADF of a typical filament region calculated from the odd and even frames in the scan. Convergence is assessed visually by the location (r, θ) of positive PADF signals, but not by quantitative comparison of the colour scale. A sufficient level of convergence to identify amorphous arcs and nanostructural arcs was achieved, and the convergence decreases at larger r values.

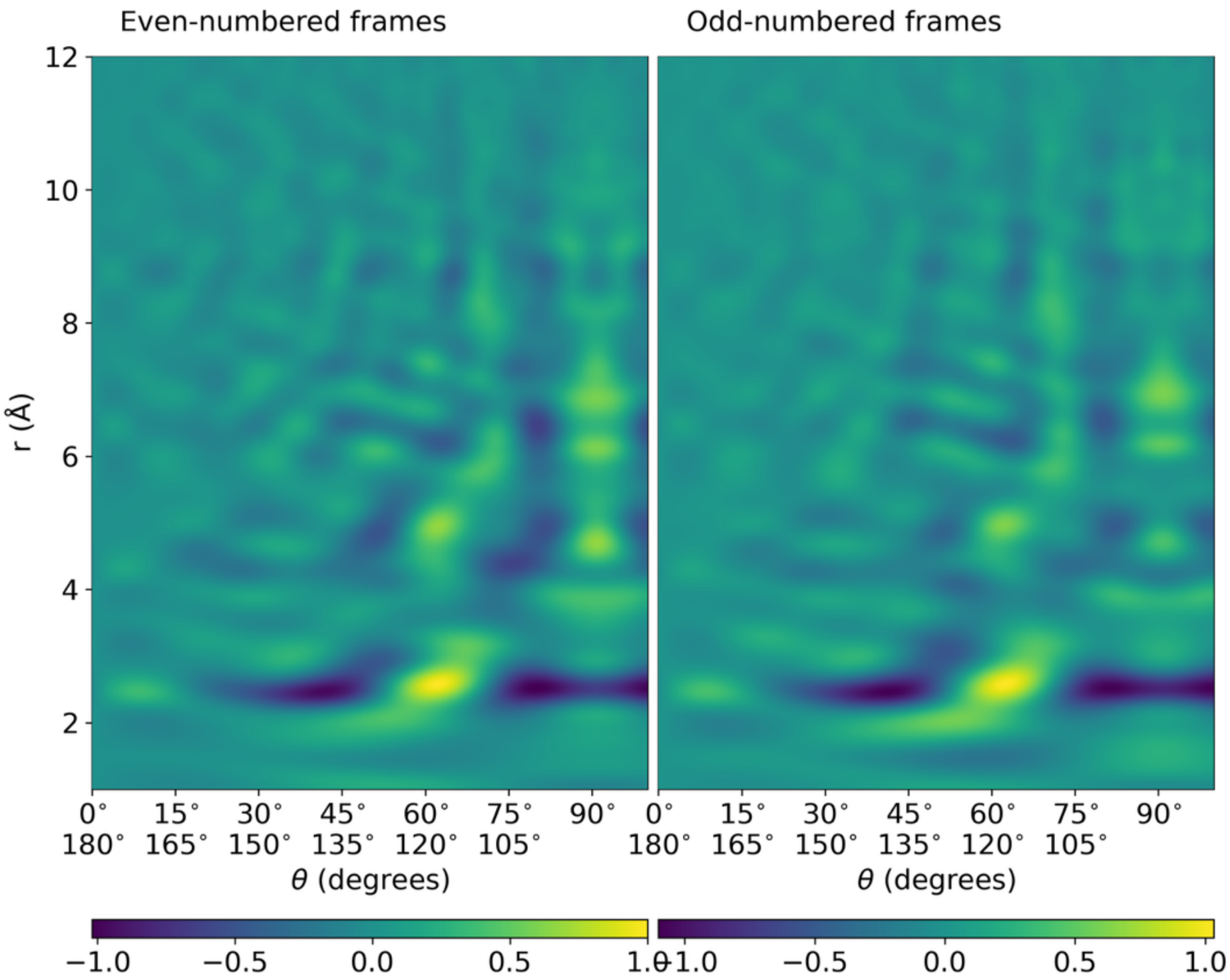

Supplementary Figure 5: Convergence test for the diamond band. The $r = r'$ slice of the PADF of a region of the diamond band calculated from the odd and even frames in the scan. Convergence is assessed visually by the location (r, θ) of positive PADF signals.

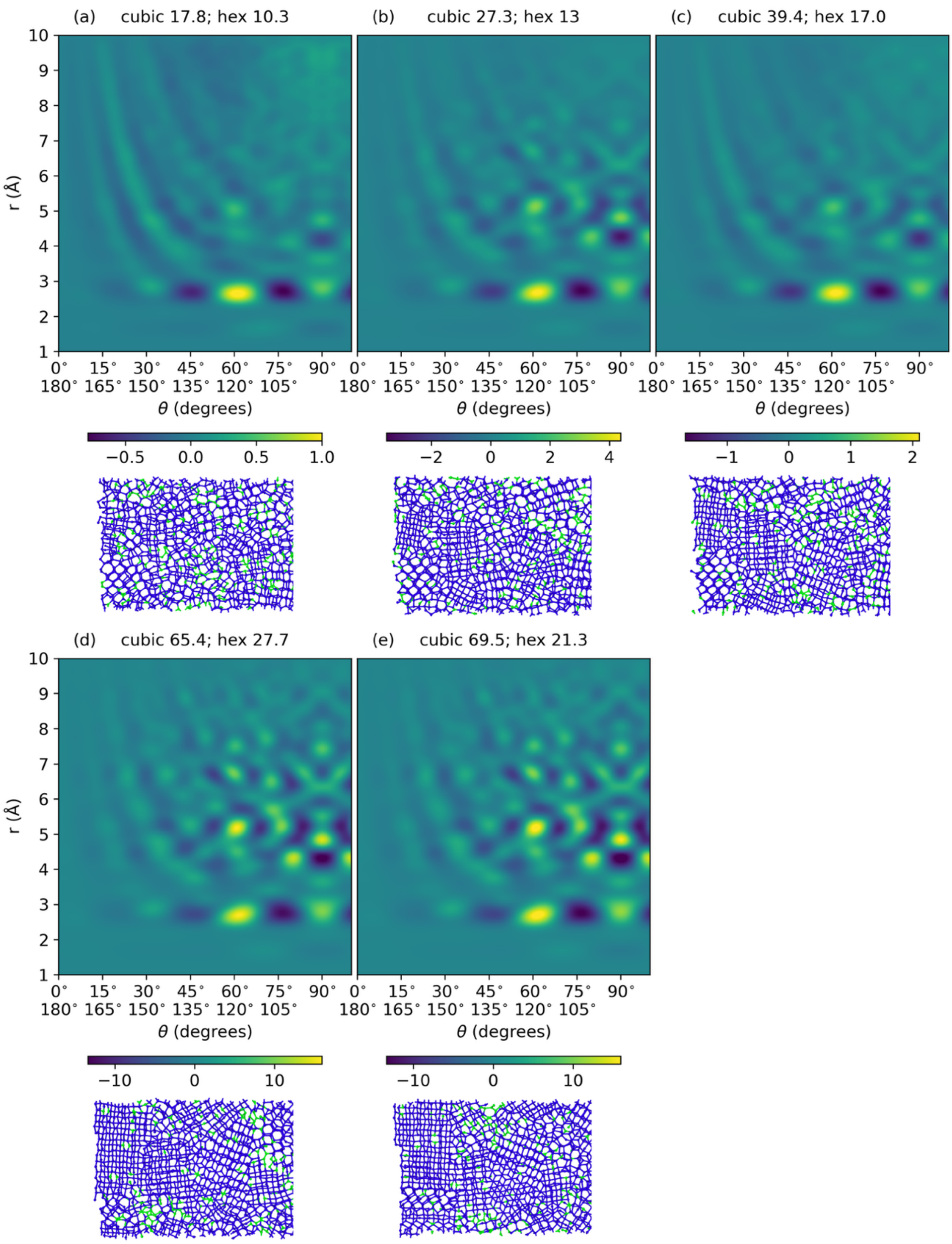

Supplementary Figure 6: Simulated PADFs of annealed nanocrystal–amorphous mixtures. The $r = r'$ slice of the PADFs of annealed structures with different ratios of crystal and amorphous structure and the corresponding structural models. The initial structure for each structure is the recovered disordered structure from the in Fig. 4 of the main text. Increasing the annealing time increased the level of cubic and hexagonal crystal structure. The title of plot indicates the percentage of cubic and hexagonal lattices present in each simulation volume. These plots show the predicted PADFs for highly ordered nanocrystals embedded in a random-network-like matrix. In the models, blue atoms are sp^3 bonded and green atoms are sp^2 bonded.

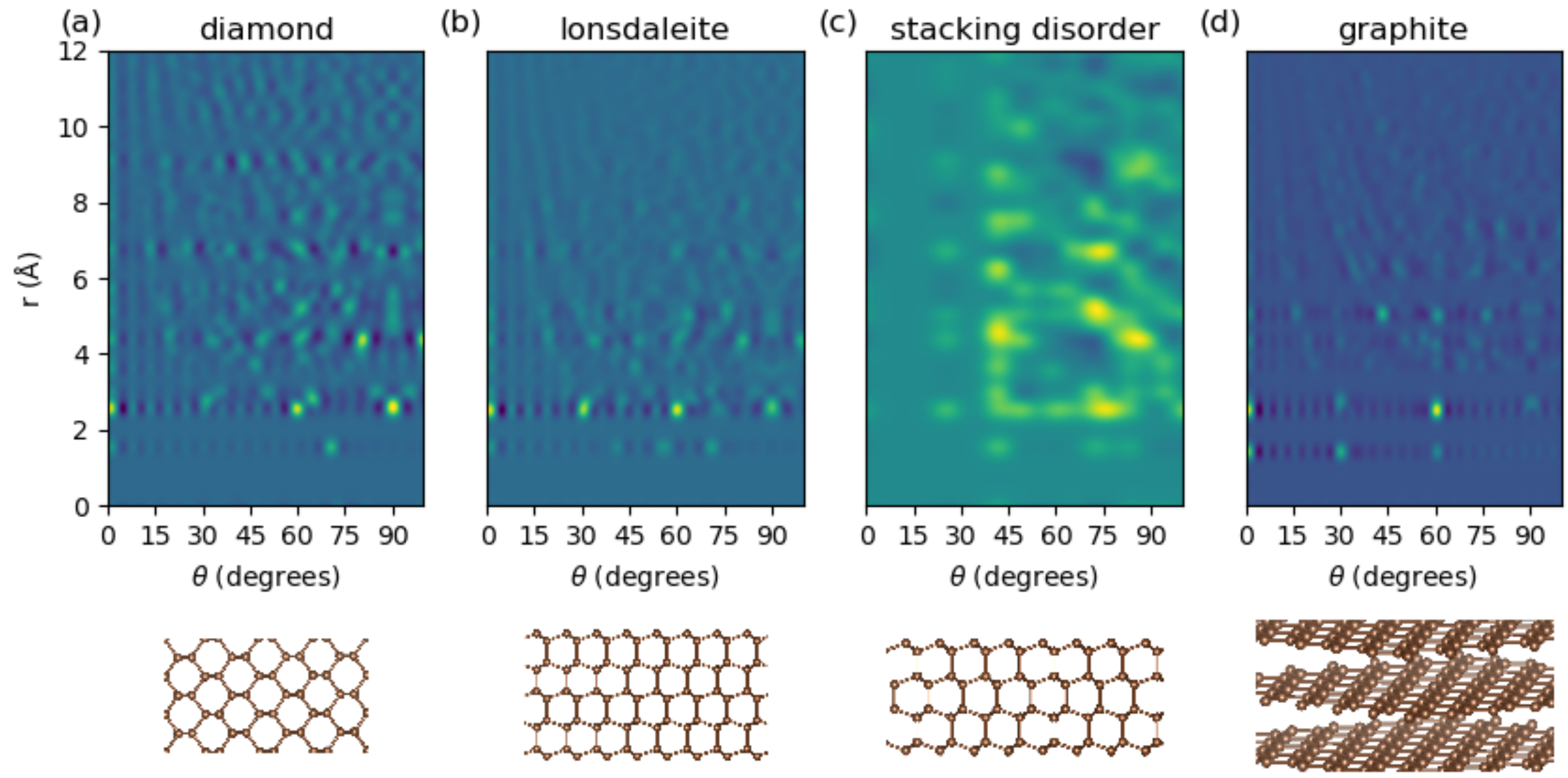

Supplementary Figure 7: Simulated PADFs of ideal and stacking-disordered carbon lattices. The $r = r'$ slice of the PADFs of (a) diamond, (b) lonsdaleite (hexagonal crystal structure), (c) stacking disordered diamond, and (d) graphite structures. These models were created from the ideal atomic positions for each lattice and the stacking disorder model is described further in the Methods of the main text.

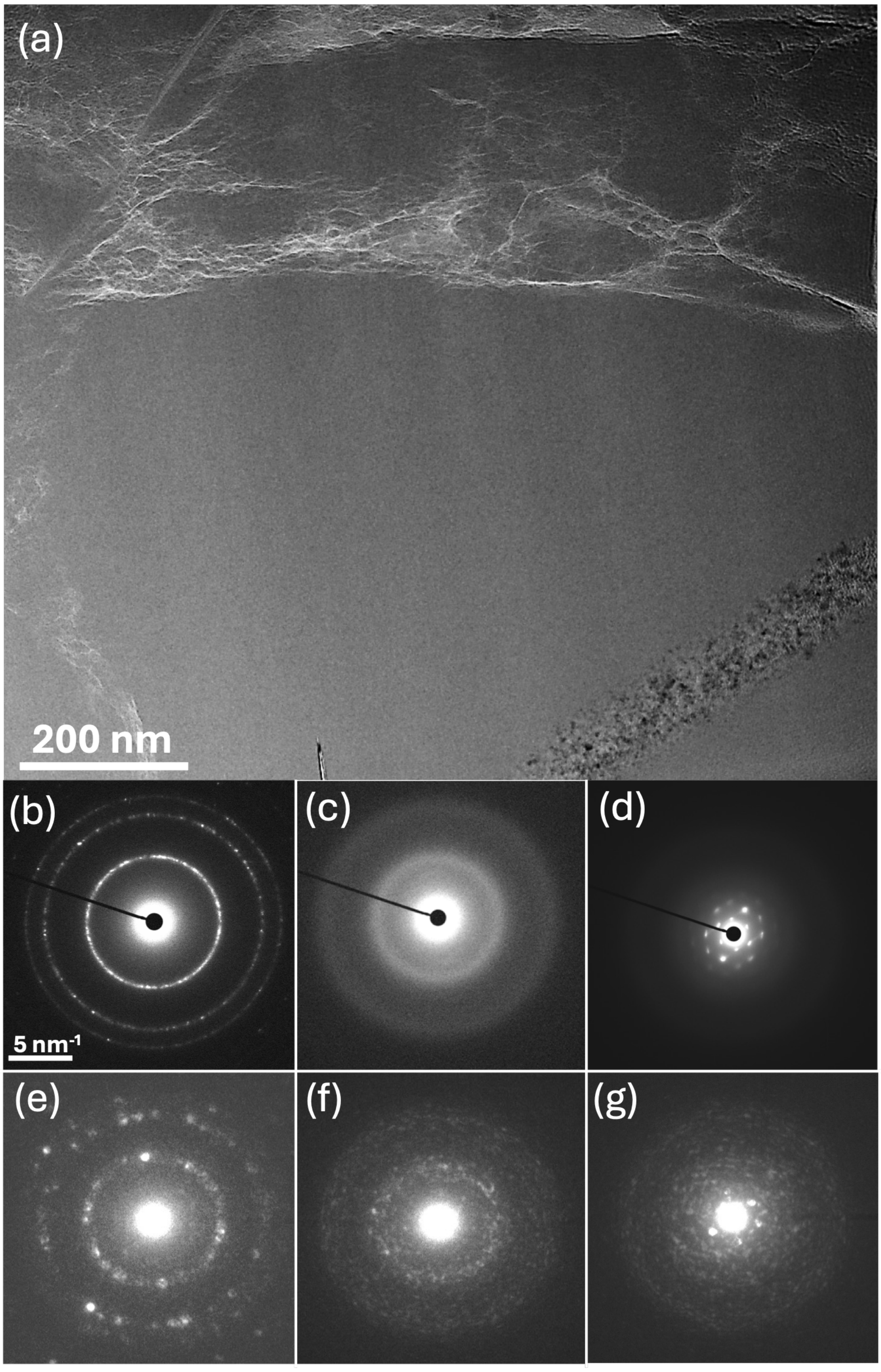

Supplementary Figure 8: **An unannotated form of Fig. 1 from the main text.** (a) A bright-field TEM image of the sample. Example selected area electron diffraction (SAED) patterns from (b) diamond band, (c) amorphous region and (d) C_{60} filament region. Equivalent example nanobeam (NB) diffraction patterns, from a 0.8 nm probe, from (e) diamond band, (f) amorphous region and (g) C_{60} filament region are shown. All SAED and nanobeam diffraction patterns are presented with the same scale.

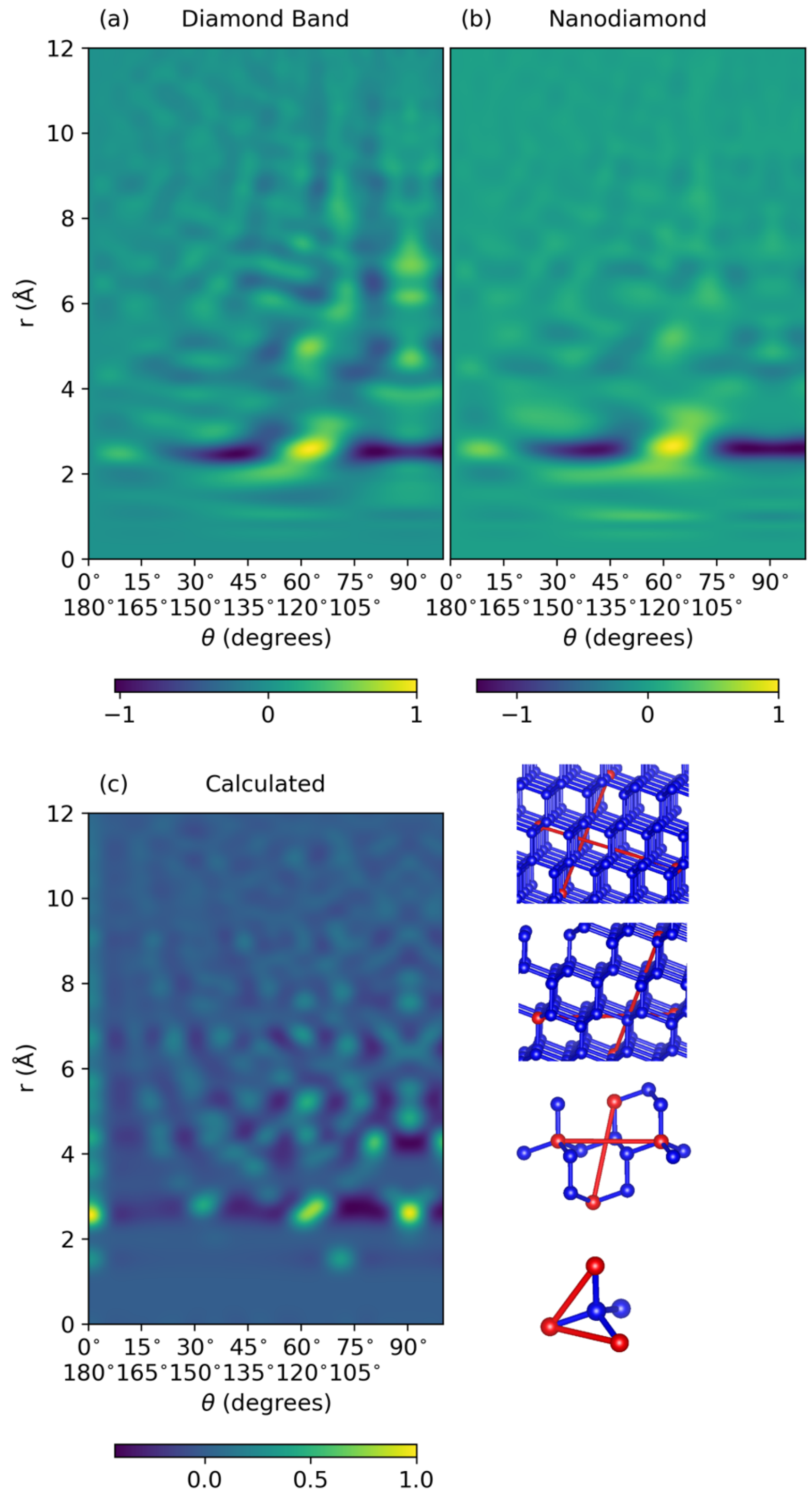

Supplementary Figure 9: **Unannotated form of Fig. 2 from the main text.** The $r = r'$ PADF slice of a diamond band in the compressed C_{60} sample. (b) A PADF of polycrystalline nanodiamonds fabricated by laser-heating glassy carbon. (c) A simulated PADF from an ideal diamond structure. Example pair combinations from the ideal diamond lattice are shown on the right-hand side of (c).

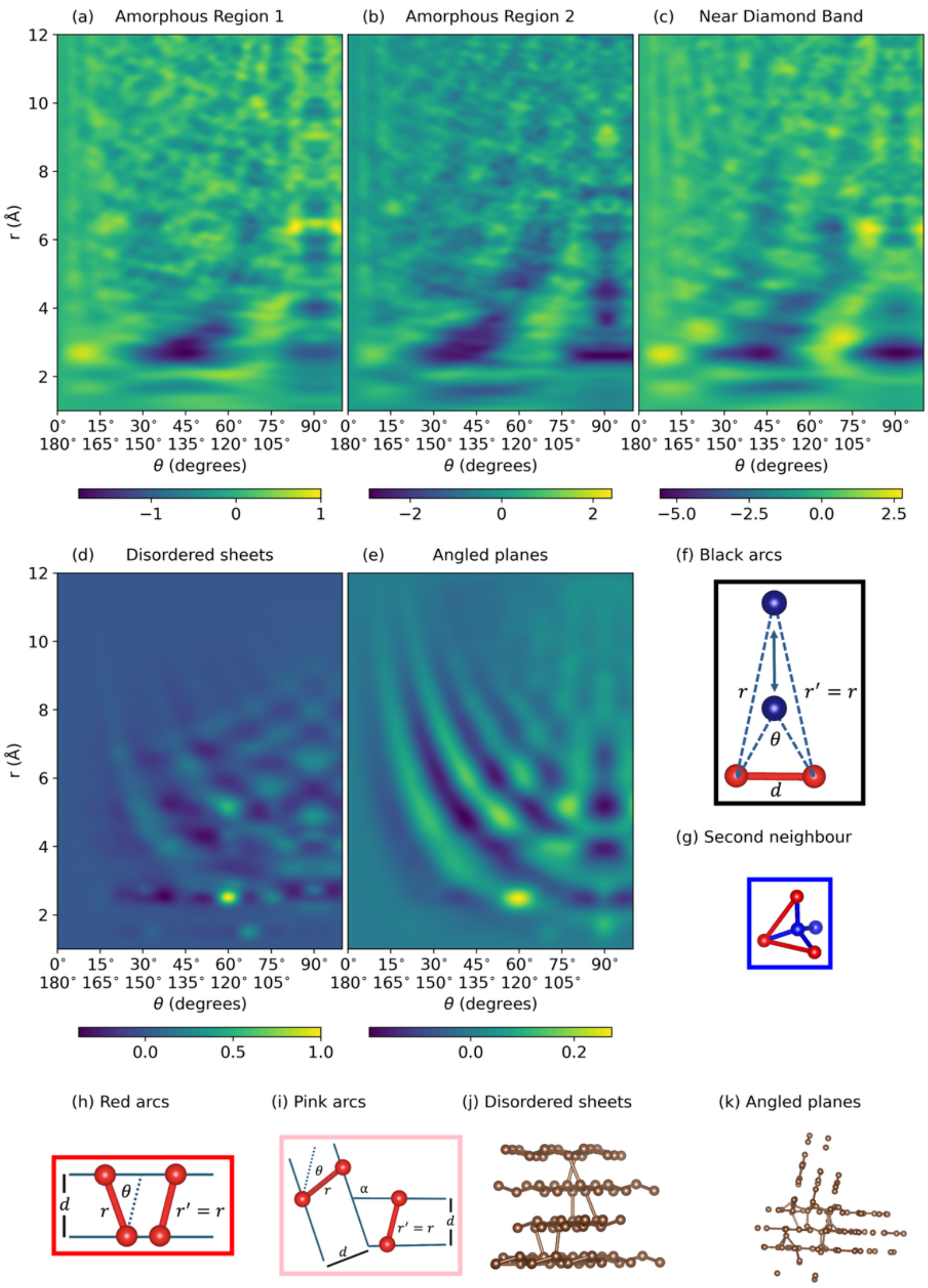

Supplementary Figure 10: Unannotated form of Fig. 3 from the main text. (a),(b) The experimental $r = r'$ cross-section of the PADFs for two different amorphous regions well separated from the diamond band, and (c) a third amorphous region close but not overlapping a diamond band. The color scale of (a) was normalised to set the maximum value to 1, and (b) & (c) were put on the same color scale. (d) The simulated PADF from randomly rotated and translated diamond layers (Disordered sheets model) and (e) from random atoms in planes at 109.5° (Angled planes model). The color scale of (d) was normalised to set the maximum value to 1, and the color scale of (e) was put on the same color scale. Structural models: (f) characteristic 2-atom pairs embedded in an amorphous structure, (g) 2nd neighbour correlation in an sp^3 bond, (h) 4-atom correlations between two disordered planes, (i) 4-atom correlations arising from sheets at a fixed angle α, (j) the disordered sheets model that matches the angular structure of Amorphous Region 1 near 90°, and (k) the angled planes model that better matches Amorphous Region 2 near 90°. In the structural models of (f)–(k), all C atoms are drawn in blue and the indicative correlated pairs are drawn in red; brown bonds in (j) and (k) are non-physical bonds introduced by the geometric construction of the model.

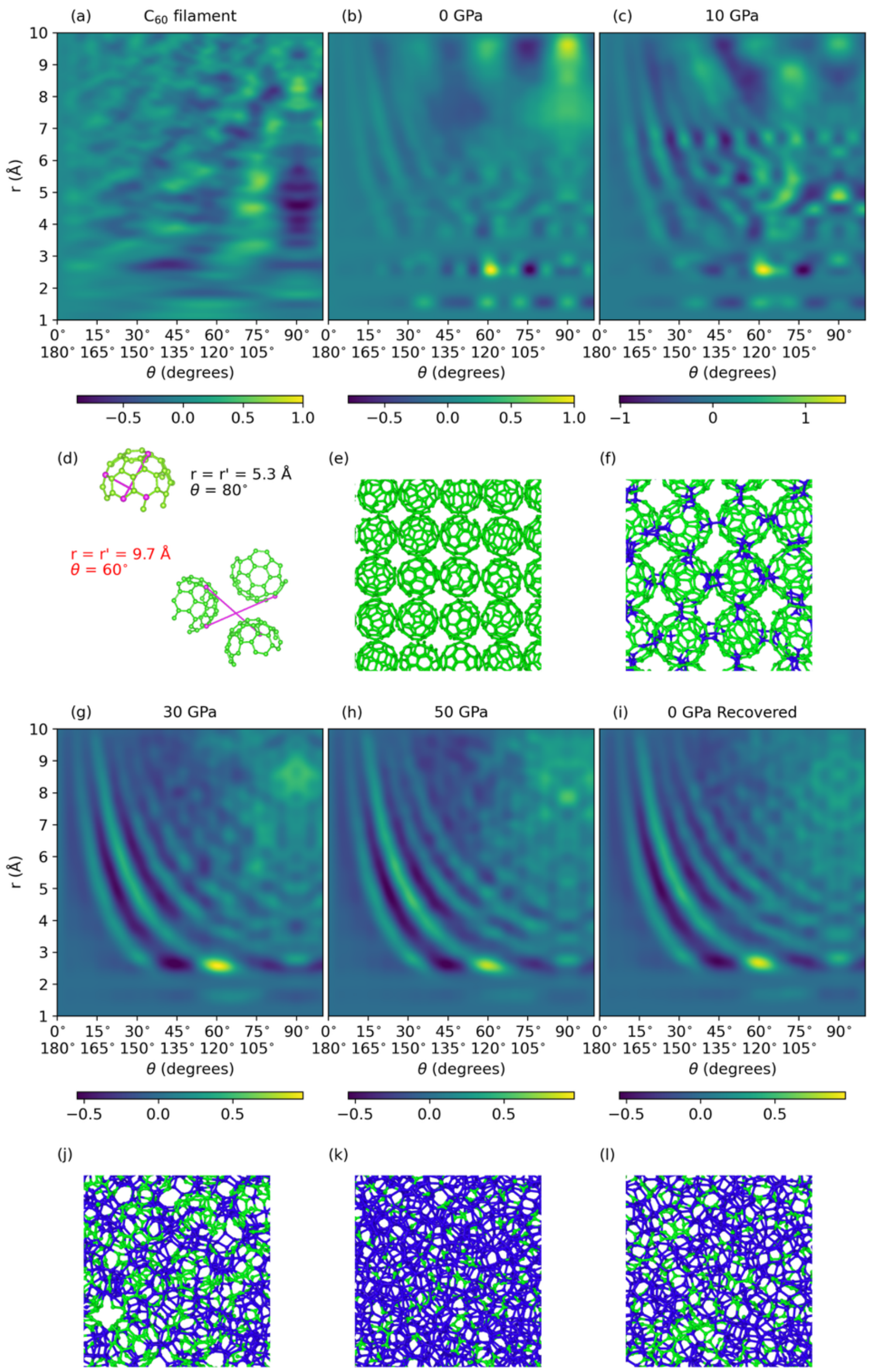

Supplementary Figure 11: Unannotated form of Fig. 4 from the main text. (a) Experimental PADF of a region with filament structures showing evidence of intact C_{60} cages. The color scale of (a) was normalised to set the maximum value to 1. (a)-(c) and (g)-(i) Simulated PADFs of a C_{60} crystal under uniaxial compression. The color scale of (b) was normalised to set the maximum value to 1, and (c),(g)-(i) were put on the same color scale. (d) Examples models from a C_{60} crystal showing key intra- and inter-cage correlations. (d)-(f),(j)-(l) Models corresponding to the simulated PADFs with sp^3 bonds shown in blue and sp^2 bonds shown in green, which show how the molecular C_{60} cages become linked and then collapse. Indicative correlation pairs are shown in magenta.
