# Supplementary material for: Towards a quantitative description of nanostructured order in amorphous diamond-like carbon
Source: Nat Commun. 2026 Sep 17;17:10938. doi: 10.1038/s41467-026-77822-4 (PMC13680357; doi:10.1038/s41467-026-77822-4)
Supplement: Supplementary file 2 [file 41467_2026_77822_MOESM2_ESM.pdf]

nature portfolio

Peer Review File

Towards a quantitative description of nanostructured order in amorphous diamond-like carbon

Corresponding Author: Dr Andrew Martin

This file contains all reviewer reports in order by version, followed by all author rebuttals in order by version.

Version 0:

Reviewer comments:

Reviewer #1

(Remarks to the Author)
This is a valuable paper that will be of broad interest because of the importance of amorphous carbon, but I have serious concerns about the way the work is presented and recommend some major changes to the structure and message of the paper.

The introductory paragraphs (and title) are overstated in that they suggest there were no experiments prior to the PADF method which have provided information beyond the PDF. Given that, I believe it is fair that the PADF is a powerful, relatively new, method which provides some directly interpretable measurements of higher order correlation functions. However they have already established this in other papers, both theoretically and experimentally with amorphous carbon. This paper is more important for what it says about the structure of certain forms of amorphous carbon rather than for advancing “Towards a quantitative description of nanostructured order in amorphous diamond-like carbon”.

I found 2 published prior references to the groups work on amorphous carbon using the PADF: “Disentangling Intra- and Interlayer Disorder in Disordered Carbons via PADF Analysis” Klemmt et. al ACS Materials Letters 2026 vol 8 p 925; and “Detection of Ring and Adatom Defects in Activated Disordered Carbon via Fluctuation Nanobeam Electron Diffraction”, Martin et. al Small, 2020, v 16 p 2000828. There are several other studies of amorphous carbon using other fluctuation diffraction based techniques which are only briefly mentioned. I think it is disingenuous to present the authors work on amorphous carbon as unprecedented, BUT I do believe their PADF method is an excellent and robust approach to obtain quantitative information on the detailed nanostructure of partially ordered regions, and this paper amplifies that with further data on carbon.
Also, other fluctuation and related 4D-STEM works have complementary places in such analysis. For example, the authors understandably use only a single probe size (0.8nm) and do not explore real-space correlation on the 1nm scale or above. For example it is well established that varying the probe size can be used to more directly measure a correlation length in Variable Resolution FEM.
The main results appear to be:
• Compressed fullerenes are strongly diamond-like amorphous structures
• There is evidence for interlinking and distortion of fullerenes in filaments after high pressure
• The work is further evidence for the value of the fluctuation diffraction techniques beyond simple pdf measurements, and f the padf in particular.

In my opinion the paper should be reorganized, including changing the title and the abstract, to emphasize the results and not the novelty of the approach, which has already been well established by the group. With these changes it could be suitable for publication in a high impact journal such as Nature Communications.

Some further minor points: the authors quote some results with HRTEM imaging of nanocrystals, but fail to point out that simple imaging without any statistical analysis is known for decades ago to be a serious drawback in structural analysis of disordered systems. Statistical analysis of image and/or diffraction data is a cornerstone of all fluctuation microscopy and diffraction techniques to overcome subjective analysis.

The authors don’t make it clear (or I missed it) that key to their method is not just a small illuminated region but a coherent probe. I assume they have verified this experimental situation.

I don’t see the three regions in the amorphous phase that they refer to in their description of Fig 1 a)? Perhaps I am misunderstanding their meaning?

(Remarks on code availability)

Reviewer #2

(Remarks to the Author)
This manuscript presents an important and timely study on the structure of non-crystalline sp3-rich carbon using the pair-angle distribution functon(PADF). The work is significant because it addresses a long-standing challenge in amorphous materials science: the lack of experimental tools capable of distinguishing a true continuous random network from disordered nanostructured phases.

A key finding of the paper is that the compressed fullerene-derived sp3-rich carbon phase is not a purely random network, but instead contains distinct short-range and medium-range order, including correlations extending from the 4th–5th neighbours (<5 Å) out to 5–10 Å. The PADF results further suggest the presence of diamond-related planes and angles, consistent with the coordination of diamond-like tetrahedral units, while also showing that the material is not sufficiently ordered to be classified as nanodiamond. This is an important result because it provides new structural insight into the nature of high-density amorphous carbon and its possible formation pathway toward diamond-like materials.

Overall, the manuscript is novel, well motivated, and suitable for publication after minor revision. I suggest the following points be addressed to improve clarity and impact:

More explanation of the PADF technique is needed. As PADF is still unfamiliar to many readers, the manuscript would benefit from a clearer explanation of how the technique works, what kind of three- and four-atom correlations it captures, and how it provides information beyond the conventional pair distribution functon(PDF).

Potential applicability to other carbon nanomaterials should be discussed. It would be useful if the authors could comment on whether PADF could also be extended to study carbon dots or carbon quantum dots, where structural disorder, nanoscale ordering, and mixed bonding environments are also highly relevant.

The intensity bars in several figures are not sufficiently clear (Figures 2, 3 and 4). The meaning of the intensity/color scales accompanying the figures should be explained more explicitly, including whether they represent normalized intensity, correlation strength, or another quantity, and whether the scales are directly comparable between figures.

In summary, this work convincingly demonstrates the power of PADF as a structural probe for amorphous and nanostructured carbon materials, and it makes a valuable contribution to the field. I support publication after minor revision.

(Remarks on code availability)
N/A

Reviewer #3

(Remarks to the Author)
This study utilizes the pair-correlation distribution function to quantitatively describe the nanostructure order in amorphous diamond-like carbon. The authors compared and analyzed the PADFs of three distinct phases in the non-crystalline sp3-rich carbon phase produced by diamond anvil cell compression of fullerenes at room, and revealed short-range and medium-range ordered nanostructures rather than the traditional random network structure, which is of great significance for understanding the structural essence of amorphous materials. Additionally, there are several issues that require authors' careful revision and clarification before publication.
1. What are the breakthrough advantages or unique analytical capabilities of the PADF technology compared to traditional characterization methods (such as Raman spectroscopy, XRD, TEM)?
2. How does PADF reveal the orientation correlation of sp2/sp3 hybridized carbon clusters? What are the limitations of traditional PDF in such analyses?
3. The PADF technology described in the manuscript is applicable to materials lacking long-range order, such as amorphous carbon. Can it be used for the characterization of the local structure of other amorphous materials? Are there any actual application cases?
4. Using a diamond anvil cell (DAC) to apply pressure to C60 powder with a micron-sized particle size for sample preparation, but without a pressure medium, how is the pressure transmitted?
5. The authors state "The convergence of the experimental PADFs is shown in the Supplementary Figs. 1-5". What is the convergence criterion?
6. The description in the first paragraph of the discussion section is somewhat confusing. For example, how can the structures shown in Fig. 4(e) and (f) be identified as CRN-like model, even though the authors have explained that "Beyond 30 GPa, the inter-molecular PADF correlations are no longer evident, and the atomic models indicate that the molecular cages collapse"? Furthermore, the CRN models used for comparison in the paper (Fig. 4(e) and (f)) are actually transformation products of C60 under high pressure from molecular dynamics simulations, rather than true CRN models. The authors need to consider selecting more appropriate CRN models to enhance the persuasiveness.

7.The color scale ranges in the figures are inconsistent. Please standardize the color scales and axes.
8.Add relevant descriptions of the figures in the supplementary information to better understand the manuscript.

(Remarks on code availability)

Version 1:

Reviewer comments:

Reviewer #1

(Remarks to the Author)
Thank-you for the several adjustments to the paper. I am fully satisfied that the paper is suitable for publication as is

(Remarks on code availability)

Reviewer #2

(Remarks to the Author)
The authors have satisfactorily addressed the reviewer's concerns. The manuscript can be accepted for publication.

(Remarks on code availability)

Open Access This Peer Review File is licensed under a Creative Commons Attribution 4.0 International License, which permits use, sharing, adaptation, distribution and reproduction in any medium or format, as long as you give appropriate credit to the original author(s) and the source, provide a link to the Creative Commons license, and indicate if changes were made.
In cases where reviewers are anonymous, credit should be given to 'Anonymous Referee' and the source.
The images or other third party material in this Peer Review File are included in the article's Creative Commons license, unless indicated otherwise in a credit line to the material. If material is not included in the article's Creative Commons license and your intended use is not permitted by statutory regulation or exceeds the permitted use, you will need to obtain permission directly from the copyright holder.
To view a copy of this license, visit https://creativecommons.org/licenses/by/4.0/

****
We thank the reviewers for all of these comments. Our responses are given below in blue text.
****

REVIEWER COMMENTS

Reviewer #1 (Remarks to the Author):

This is a valuable paper that will be of broad interest because of the importance of amorphous carbon, but I have serious concerns about the way the work is presented and recommend some major changes to the structure and message of the paper.

The introductory paragraphs (and title) are overstated in that they suggest there were no experiments prior to the PADF method which have provided information beyond the PDF. Given that, I believe it is fair that the PADF is a powerful, relatively new, method which provides some directly interpretable measurements of higher order correlation functions. However they have already established this in other papers, both theoretically and experimentally with amorphous carbon. This paper is more important for what it says about the structure of certain forms of amorphous carbon rather than for advancing “Towards a quantitative description of nanostructured order in amorphous diamond-like carbon”.
****
We appreciate this point by the reviewer. As it is related to the next reviewer’s paragraph, please see our response below.
****

I found 2 published prior references to the groups work on amorphous carbon using the PADF: “Disentangling Intra- and Interlayer Disorder in Disordered Carbons via PADF Analysis” Klemmt et. al ACS Materials Letters 2026 vol 8 p 925; and “Detection of Ring and Adatom Defects in Activated Disordered Carbon via Fluctuation Nanobeam Electron Diffraction”, Martin et. al Small, 2020, v 16 p 2000828. There are several other studies of amorphous carbon using other fluctuation diffraction based techniques which are only briefly mentioned. I think it is disingenuous to present the authors work on amorphous carbon as unprecedented, BUT I do believe their PADF method is an excellent and robust approach to obtain quantitative information on the detailed nanostructure of partially ordered regions, and this paper amplifies that with further data on carbon.
****
We appreciate the reviewer’s point, and we
1) agree that we could cover prior fluctuation studies in the introduction, but
2) respectfully disagree that we have published on “amorphous carbon” previously.

Our previous studies were on sp^2-bonded activated carbons and hard (battery) carbons, which are considered non-graphitic carbons and not amorphous carbons. This distinction here is that the activated & hard carbons display clear nano-scale planar structures in TEM images and other techniques, which amorphous carbons do not. The classification about the naming of carbon materials has been recently addressed in the literature, supporting our use of this terminology in our manuscript: see Glatthaar et al. Angew Chem Int Ed Engl 2026 Vol. 65 Issue 11 Pages e19975.

We appreciate that the distinction between amorphous and disordered carbons may not be clear to readers who are less familiar with recent carbon literature. We have added the following text to clarify this point to page :

"*Both the activated carbons and sp^2-hybridised disordered carbons have locally graphitic structure that is clearly observable in high resolution TEM imaging, and neither is considered an amorphous carbon[36]. To date there have been no reported PADF measurements of an amorphous material to our knowledge.*"

We have added the following reference: Ref. 36: by Glatthaar et al. Angew Chem Int Ed Engl 2026 Vol. 65 Issue 11 Pages e19975.

To better acknowledge the prior work on fluctuation studies of amorphous materials including amorphous carbon, and how our PADF studies extend upon it, we have added the following paragraph to the introduction on page 3:

"Fluctuation electron microscopy (FEM) and fluctuation diffraction are statistical techniques with a demonstrated sensitivity for probing medium-range order in amorphous materials[1,25] and have been used to support paracrystalline models[12,15]. An extensive FEM study of several carbon materials by Zhao et al.[25] showed the sensitivity of FEM to local structures in disordered graphitic carbons and fullerenes. Zhao et al. included a tetrahedral amorphous carbon in their study, which yielded a weak FEM signal interpreted as a CRN-like structure. Despite these results, fluctuation microscopy and fluctuation diffraction are reliant on available models for structural interpretation and have not fully resolved structural debates concerning paracrystals and CRN structures[26]. They are yet to be widely adopted as a standard characterisation technique for amorphous materials. This motivates the development of new approaches to extract real-space structural information from fluctuation data."

We have added the following reference
[26] Roorda, S. *et al. Science* **338**, 1539 (2012)
****

Also, other fluctuation and related 4D-STEM works have complementary places in such analysis. For example, the authors understandably use only a single probe size (0.8nm) and do not explore real-space correlation on the 1nm scale or above. For example it is well established that varying the probe size can be used to more directly measure a correlation length in Variable Resolution FEM.

****
This is an excellent point. We do think other techniques are complementary. We have added the following section to the discussion on page 6 to explain the effect of probe size on the PADF and to communicate the complementary use of PADF and variable-resolution FEM:

"There are opportunities to access larger distance scales in the PADF by increasing the probe size beyond the 0.8 nm used in this study. Our results show structural information up to 1.2 nm, suggesting that signal is measured from the outer parts of

the beam or some spreading of the probe in the sample. Increasing the beam size increases the largest r value accessible in the PADF, but also creates a larger interaction volume which may require more diffraction data for convergence. It is unknown how the number of patterns will scale with probe size. The PADF is complementary to existing statistical measures, such as variable-resolution FEM that collect FEM signals as a function of probe size[41]. The comparison of PADF and variable-resolution FEM would corroborate both statistical significance and structural interpretation."

We have added the reference:
[41] Bogle, S. N. *et al. Ultramicroscopy* **110**, 1273 (2010).

****

The main results appear to be:
• Compressed fullerenes are strongly diamond-like amorphous structures
• There is evidence for interlinking and distortion of fullerenes in filaments after high pressure
• The work is further evidence for the value of the fluctuation diffraction techniques beyond simple pdf measurements, and f the padf in particular.

In my opinion the paper should be reorganized, including changing the title and the abstract, to emphasize the results and not the novelty of the approach, which has already been well established by the group. With these changes it could be suitable for publication in a high impact journal such as Nature Communications.

****
We have carefully considered the reviewer's suggestion to change the title and the abstract. However, in light of our response above about the difference between non-graphitising carbon materials and amorphous carbons, we consider the current title and abstract to have the right emphasis on the problem of amorphous carbon structure, which our previous studies of disordered carbons do not address. It's only by emphasising the successful application of the PADF to a genuinely amorphous structure that we can highlight the opportunities provided by the PADF to address other long-standing problems in the structure of amorphous silicon, amorphous oxides, metallic glasses and more.
****

Some further minor points: the authors quote some results with HRTEM imaging of nanocrystals, but fail to point out that simple imaging without any statistical analysis is known for decades ago to be a serious drawback in structural analysis of disordered systems. Statistical analysis of image and/or diffraction data is a cornerstone of all fluctuation microscopy and diffraction techniques to overcome subjective analysis.
****
This is an excellent point raised by the reviewer. The follow text has been added at the end of page 2 and beginning of page 3:

"*It is critical that new characterisation techniques are based on a detailed statistical analysis of structure, which can infer the properties of the bulk material, and not the*

subjective analysis of individual TEM images. This motivates the development of characterisation techniques capable of probing statistically significant structure beyond average short-range order."
****

The authors don't make it clear (or I missed it) that key to their method is not just a small illuminated region but a coherent probe. I assume they have verified this experimental situation.

****
We find the reviewer's comment that coherence is the "key" to the PADF technique is a bit misleading, because it implies that the PADF technique requires more coherence than other standard electron diffraction techniques. This is not true. Our method requires the same amount of beam coherence as crystallography, PDF analysis or common TEM imaging. This is based on standard diffraction theory, in which the interference observed in the diffraction pattern requires some level of beam coherence. The coherence length needs to be larger than the structural length scale of interest, in this case about ~1-2nm, which it typically is in most systems.

Typically coherence is stated as a requirement of diffraction techniques that require full beam coherence, such as coherent diffractive imaging or fluctuation-based 3D imaging of single particles by X-ray free-electron lasers. The PADF technique does not require the same level of beam coherence as these techniques. To emphasise that the PADF technique has no special coherence requirements and is accessible on most high-end TEM systems we have added the following statement to page 3 of the paper:

"PADF measurements can be performed on TEMs with a nanobeam diffraction capability and it has similar coherence requirements to other standard electron diffraction techniques"
****

I don't see the three regions in the amorphous phase that they refer to in their description of Fig 1 a)? Perhaps I am misunderstanding their meaning?

****
We don't have an image that shows the exact locations of amorphous regions 1 & 2, but their separation from the filament regions and the diamond band region is similar to that shown by the indicated amorphous region in Fig. 1. This was already clarified in the caption to Fig. 1 by the following text:

"Note the amorphous region is not the exact measurement location, but is indicative of the separation of the measured amorphous regions (labelled Amorphous Region 1 and Amorphous Region 2 in later figures) from the non-amorphous regions."
****

Reviewer #2 (Remarks to the Author):

This manuscript presents an important and timely study on the structure of non-crystalline sp3-rich carbon using the pair-angle distribution functon(PADF). The work is significant because it addresses a long-standing challenge in amorphous materials science: the lack of experimental tools capable of distinguishing a true continuous random network from disordered nanostructured phases.

A key finding of the paper is that the compressed fullerene-derived sp3-rich carbon phase is not a purely random network, but instead contains distinct short-range and medium-range order, including correlations extending from the 4th–5th neighbours (<5 Å) out to 5–10 Å. The PADF results further suggest the presence of diamond-related planes and angles, consistent with the coordination of diamond-like tetrahedral units, while also showing that the material is not sufficiently ordered to be classified as nanodiamond. This is an important result because it provides new structural insight into the nature of high-density amorphous carbon and its possible formation pathway toward diamond-like materials.

Overall, the manuscript is novel, well motivated, and suitable for publication after minor revision. I suggest the following points be addressed to improve clarity and impact:

More explanation of the PADF technique is needed. As PADF is still unfamiliar to many readers, the manuscript would benefit from a clearer explanation of how the technique works, what kind of three- and four-atom correlations it captures, and how it provides information beyond the conventional pair distribution functon(PDF).

****

This is a good suggestion for readers who are not familiar with the previous PADF literature. Since mathematical details of the PADF have been extensively covered in the literature, we have added a brief explanation to the methods and further citations to the literature. On page 7 of the manuscript we have added the following text:

"The PADF is an atomic correlation function that includes contributions from three- and four-atom correlations. It is a function of two pair distances (r and r') and an angle between the pairs (θ). It is computed from the angular cross-correlation function of a scanning nanobeam diffraction dataset. The mathematical details of the intensity correlation function[45,46], the PADF[25] and the transformation to produce the PADF[26] can be found in the cited literature."

We have added the following two references for further details about intensity correlation functions:

45. Kurta, R. P. et al. Structural Analysis by X-ray Intensity Angular Cross Correlations. Vol. 161 (2016).

46. Kam, Z. Macromolecules 10, 927 (1977)

****

Potential applicability to other carbon nanomaterials should be discussed. It would

be useful if the authors could comment on whether PADF could also be extended to study carbon dots or carbon quantum dots, where structural disorder, nanoscale ordering, and mixed bonding environments are also highly relevant.

****
We thank the reviewer for this suggestion. Future applications of the PADF technique to carbon dots are feasible and we have added the following text on page 6 of the manuscript:
"Beyond bulk materials, there are further opportunities to apply the PADF technique to complex nanoparticle structures such as carbon dots[40]."

A new reference has been added:
[40] Li, H. et al. Journal of Materials Chemistry 22, 24230 (2012)
****

The intensity bars in several figures are not sufficiently clear (Figures 2, 3 and 4). The meaning of the intensity/color scales accompanying the figures should be explained more explicitly, including whether they represent normalized intensity, correlation strength, or another quantity, and whether the scales are directly comparable between figures.

****
Statements have been added to each caption of Figs, 2, 3 and 4 to explain the colour scale of each PADF measurement.
****

In summary, this work convincingly demonstrates the power of PADF as a structural probe for amorphous and nanostructured carbon materials, and it makes a valuable contribution to the field. I support publication after minor revision.

Reviewer #2 (Remarks on code availability):

N/A

Reviewer #3 (Remarks to the Author):

This study utilizes the pair-correlation distribution function to quantitatively describe the nanostructure order in amorphous diamond-like carbon. The authors compared and analyzed the PADFs of three distinct phases in the non-crystalline sp3-rich carbon phase produced by diamond anvil cell compression of fullerenes at room, and revealed short-range and medium-range ordered nanostructures rather than the traditional random network structure, which is of great significance for understanding the structural essence of amorphous materials. Additionally, there are several issues that require authors' careful revision and clarification before publication.

1. What are the breakthrough advantages or unique analytical capabilities of the PADF technology compared to traditional characterization methods (such as Raman spectroscopy, XRD, TEM)?

****

We agree with the reviewer that this is an important point. It was addressed in the original manuscript in the introduction by the text on page 3:
"This overcomes the limitations of other methods such as projection TEM imaging, which rely heavily on paracrystalline or nanocrystalline models to detect nanostructure. It also overcomes the limitation of established pair distribution methods that lack local angular information."

To further reiterate this point in the conclusion, we have added to the top of page 7:
"It contains key angular information in real-space that is not accessible by conventional electron or x-ray diffraction, PDF analysis, or spectroscopy."

We view that these statements in the text achieve a balance between highlighting the advantages of the PADF, without making overly generalised claims about the limitations of existing techniques.
****

2. How does PADF reveal the orientation correlation of sp2/sp3 hybridized carbon clusters? What are the limitations of traditional PDF in such analyses?

****

The potential role of sp2/sp3 hybridization in the disordered nanostructures is a very interesting point that the PADF does not reveal directly without the help of further modelling. To clarify this in the manuscript we have added the following text to the discussion on page 6 of the manuscript:

"*A key question is the role of sp^2 and sp^3 bonds in these disordered nanostructures. Since the PADF correlations of these nanostructures do not resemble graphene or nanoscale graphite, they are unlikely to be purely sp^2 bonded. It is possible that hybridisation of sp^2 and sp^3 bonds is key to understanding their disordered nature, which could be quantified by further modelling of the nanostructures that agree with the experimental PADF measurements."*

Since we have not quantified the hybridisation in our study, it is premature to comment on the relative limitations of the PADF vs PDF techniques on this point.
****

3. The PADF technology described in the manuscript is applicable to materials lacking long-range order, such as amorphous carbon. Can it be used for the characterization of the local structure of other amorphous materials? Are there any actual application cases?

****
We agree that this is an important point to highlight, but on reviewing the manuscript we consider that it was already adequately covered in our concluding paragraph on page 7 by the following text:
"*The PADF technique has future applications to glasses, metallic glasses, and other amorphous solids could lead to a fundamentally deeper understanding of amorphous structure and its relationship to material properties. Beyond characterisation, the physical insight gained through PADF analysis can directly inform synthesis strategies. In the present case, the rotational and angular correlations revealed by the PADF further confirms that non-hydrostatic compression, or shear conditions, favour the formation of diamond-like tetrahedral coordination networks. This points toward concrete experimental pathways for the targeted synthesis of new amorphous diamond-like materials."*
****

4. Using a diamond anvil cell (DAC) to apply pressure to C60 powder with a micron-sized particle size for sample preparation, but without a pressure medium, how is the pressure transmitted?

****
The technique of using a DAC without a pressure medium is well-established in our previous publications [McCulloch et al. *Small,* 16, 2004695 (2020), Heimes et al. Diamond and Rel. Mater. 159, 112776 (2025)] and also those of other groups [Lai, S., et al. Nature 651, 621–625 (2026)]. Briefly, without a pressure medium the pressure transmission is non-hydrostatic, deviatoric (shear) stresses develop between grain contacts as the cell is closed, and pressure gradients exist across the sample chamber. This is a deliberate and consequential aspect of our experimental design. Non-hydrostatic loading is well-established to lower phase transformation boundaries relative to hydrostatic conditions, due to the additional mechanical driving force provided by shear stress. This promotes transformations that may be kinetically or thermodynamically suppressed under purely hydrostatic compression.

To assist the reader to finding this literature if they wish to learn more about the technique, we have added the following text to the Methods on page 7 of the manuscript:

"*Further details about the DAC technique without a pressure medium can be found in McCulloch et al.*[42] *and Liu et al.* [43]"

We have added the new reference
[42] McCulloch et al. *Small,* 16, 2004695 (2020)
[43] Lai, S., et al. Bulk hexagonal diamond. Nature 651, 621–625 (2026)
****

5. The authors state "The convergence of the experimental PADFs is shown in the Supplementary Figs. 1-5". What is the convergence criterion?

****
Text has been added to each caption in Supplementary Figs. 1-5 to clarify the convergence. For example, the text added to the caption of Supp. Fig. 1 is:

"Convergence is assessed visually by the location (r, θ) of positive PADF signals, but not by quantitative comparison of the colour scale. A sufficient level of convergence to identify amorphous arcs and nanostructural arcs was achieved, and the convergence decreases at larger r values."
****

6. The description in the first paragraph of the discussion section is somewhat confusing. For example, how can the structures shown in Fig. 4(e) and (f) be identified as CRN-like model, even though the authors have explained that "Beyond 30 GPa, the inter-molecular PADF correlations are no longer evident, and the atomic models indicate that the molecular cages collapse"? Furthermore, the CRN models used for comparison in the paper (Fig. 4(e) and (f)) are actually transformation products of C60 under high pressure from molecular dynamics simulations, rather than true CRN models. The authors need to consider selecting more appropriate CRN models to enhance the persuasiveness.

****
We appreciate the reviewer's point on the technical difference between true CRN models and MD models of highly disordered structure, and took care in the manuscript to refer to our models as "CRN-like" rather than "CRNs". The models of transformation products display the expected PADF signatures of a material with only short-range order, which are also expected of true CRN structures, and sufficient to support our claims in the paper. To clarify the expected PADF structure of an ideal CRN model we have added the following comment to page 6 of the manuscript:

"An ideal CRN model is expected to have similar or less angular order to the simulated structures in Figs. 4(e) and (f), including the 1st and 2nd neighbour peaks, the amorphous arcs, and lack of medium-range order."

7.The color scale ranges in the figures are inconsistent. Please standardize the color scales and axes.

****
A similar comment was made by Reviewer 2. As per the response to reviewer 2, the captions of Figs. 2-4 have been updated to explain the normalisation of the colour scale for each figure. The colour scale of comparable PADFs had already been put

on the same scale where appropriate (i.e. experimental and simulated PADF may be normalised independently).
****

8.Add relevant descriptions of the figures in the supplementary information to better understand the manuscript.

****
Supplementary Figures 1-5 have had text added to the caption about convergence, as noted above.

To supplementary Fig. 6 we have added the following explanation to the caption:
"The initial structure for each structure is the recovered disordered structure from the in Fig. 4 of the main text. Increasing the annealing time increased the level of cubic and hexagonal crystal structure."

To Supplementary Fig. 7 we have added the following text:
"These models were created from the ideal atomic positions for each lattice and the stacking disorder model is described further in the Methods of the main text."
****
